# Assessing the impact of contraceptive use on reproductive cancer risk among women of reproductive age—a systematic review

**DOI:** 10.3389/fgwh.2024.1487820

**Published:** 2024-11-13

**Authors:** Shayesteh Jahanfar, Julie Mortazavi, Amy Lapidow, Cassandra Cu, Jude Al Abosy, Kathyrn Morris, Juan Camilo Becerra-Mateus, Meredith Steinfeldt, Olivia Maurer, Jiang Bohang, Paola Andrenacci, Marwa Badawy, Moazzam Ali

**Affiliations:** ^1^ Department of Public Health and Community Medicine, Tufts University School of Medicine, Boston, MA, United States; ^2^ School of Medicine, Tufts University School of Medicine, Boston, MA, United States; ^3^ Department of Public Health and Community Medicine, Universidad de Antioquia, Medellin, Columbia; ^4^ Cochrane US Mentoring Program, Boston, MA, United States; ^5^ US Mentoring Program, Boston, MA, United States; ^6^ WHO Department of Sexual and Reproductive Health and Research, World Health Organization, Geneva, Switzerland

**Keywords:** contraceptives, ovarian cancer, cervical cancer, endometrial cancer, breast cancer

## Abstract

**Background:**

Contraceptives play a crucial role in women's reproductive health, their hormonal components may be linked to cancer risks, specifically breast, and gynecological cancers. Given the high usage rates of hormonal contraceptives, it is vital to systematically evaluate their potential impact on cancer outcomes, especially among women with a family history of gynecological cancers.

**Objectives:**

This study aims to evaluate the evidence on the association between modern contraceptive use and the risk of breast and reproductive cancers (ovarian, endometrial, and cervical cancer) among women of reproductive age, to inform healthcare providers, women, and program managers about cancer outcomes related to contraceptive use.

**Methods:**

A systematic review was conducted according to PRISMA guidelines. Searches were performed in databases such as CINAHL, OVID Medline, EMBASE, and more from inception to February 2022. Eligible studies included randomized controlled trials, cohort studies, and case-control studies that compared cancer outcomes between contraceptive users and non-users. Data extraction, quality assessment, and meta-analyses were conducted following predefined protocols. Subgroup and sensitivity analyses examined variations in contraceptive methods, doses, and duration.

**Results:**

A total of 51 studies were included, comprising 2 RCTs and 49 observational studies. The review identified a significant reduction in ovarian and endometrial cancer incidence among contraceptive users. Hormonal contraceptive users had a 36% lower risk of ovarian cancer (RR 0.64, 95% CI 0.60–0.68), with specific reductions seen in combined oral contraceptive users (RR 0.62, 95% CI 0.57–0.68) and hormonal IUD users (RR 0.68, 95% CI 0.48–0.96). The rate ratio of cervical cancer was higher among non- users compared to hormonal contraceptive users when we pooled the results (1.28, 95% CI 1.21, 1.35). No significant association was found between contraceptive use and breast cancer risk among healthy women (RR 1.00, 95% CI 0.94–1.06). However, BRCA1/2 mutation carriers using oral contraceptives showed a heightened risk of breast cancer (HR 1.39, 95% CI 1.15–1.67).

**Conclusion:**

This systematic review highlights the protective effects of modern contraceptives against ovarian and endometrial cancers while identifying an increased risk of cervical. No significant breast cancer risk was found for healthy women, but BRCA1/2 mutation carriers faced increased risks. These findings underscore the need for personalized contraceptive counselling that considers cancer risk factors. Further research is needed to explore contraceptive impacts across different genetic profiles and dosing regimens.

**Systematic Review Registration:**

https://www.crd.york.ac.uk/prospero/, Prospero (CRD42022332647).

## Background

Over the last two decades, the number of women using a modern contraceptive increased from 663 million to 851 million. By the year 2030, it is projected that an additional 70 million women will be using contraception (1). Modern contraceptives have therefore become an integral part of women's health globally.

Modern contraceptives come in three forms: short-acting, long-acting, and one-time barrier. Short-acting contraceptives include oral contraceptive pills (OCPs) (151 million users, 16%), injections (74 million users, 8%), and patches and vaginal rings (less than 15 million users, less than 2%). Long-acting contraceptives include intrauterine devices (IUDs) (159 million users, 17%), implanted devices (23 million users, 2%), and female sterilization (219 million users, 24%). One-time barrier contraceptives include sponges, diaphragms, cervical caps, spermicide, female condoms, and male condoms. Research shows that global use for all one-time barrier methods except for male condom use (189 million users, 21%) is low (1).

Hormonal methods of contraception contain either a combination of estrogen and progestin, or progestin only. Reproductive hormones of the ovaries, estrogen and progesterone, play a critical role in the genesis of breast, and gynecological cancers, as these two hormones are carcinogens (2). Worldwide, breast cancer are the top two most common cancer incidence in women following gynecological cancers: cervical 4th, endometrial/uterine 6th, and ovary 8th most common cancers (3).

Given the potential links between modern contraceptive use and cancer risk, it is critical for women, health care providers, and program managers to be well-informed about the benefits and risks of each method. The current medical eligibility criteria for safe contraceptive use does not have guidelines for women with a family history of gynecological cancers (see Supplementary Appendix 1 - A) (4). Despite the widespread use of contraceptives, guidelines for women with a family history of gynecological cancers are lacking. This study seeks to systematically evaluate evidence regarding the impact of contraceptives on cancer outcomes among women of reproductive age, emphasizing key factors influencing reproductive cancer risk. The review attempts to enhance awareness among women, healthcare providers, and program managers, facilitating informed decision-making regarding contraceptive choices in the context of cancer risks. This study aims to identify and evaluate evidence that focuses on the use of contraceptives and their impact on cancer outcomes among women of reproductive age. There are three factors that we considered in connection to reproductive cancer risk throughout this review: Contraceptive use, genetic background and lifestyle factors.

## Methods

This systematic review is registered on Prospero (CRD42022332647). It is focused on quantitative evidence with reportable outcomes and measures of risk regarding the relationship between the use of contraceptives and cancer morbidity and mortality among women of reproductive age. This review also focused on evidence that is consistent within a specific population, intervention, comparison, outcome, and study design (PICO) or population, exposure, comparison, outcome and study design (PECO). The population of interest was women of reproductive age (14–49 years of age). When drawing data from interventional studies, all modern contraceptive methods that the WHO defines as effective and acceptable were included. Such methods include (1) short-acting hormonal contraception (e.g., OCPs, patches, and vaginal rings), (2) long-term contraception (e.g., hormonal or non-hormonal IUD, implants, and injections), (3) one-time barrier contraception (e.g., condoms, sponges, diaphragms, cervical caps, and spermicide), (4) permanent contraception (e.g., tubal ligation and vasectomy), and (5) emergency contraception (e.g., morning after pill or IUD). Contraceptive use of all types (as stated above) was considered the main exposure for observational studies. Any other study that investigated contraception in conjunction with other medications or modalities was excluded. We included studies whose comparison group consisted of non-users of contraception only. The outcome of interest was mortality and morbidity due to breast eproductive tract cancers (e.g., ovarian cancer, endometrial cancer, and cervical cancer). We included studies with any one of the following study designs: parallel or cluster randomized controlled trials (RCTs), controlled clinical trials, controlled before and after studies, interrupted time series studies, cohort or longitudinal analyses, regression discontinuity designs, and case- control studies.

To avoid publication bias, we searched for published or unpublished studies from inception to February 2022, with no language or geographical boundaries. Inception dates vary by database; we searched the CINAHL, OVID Medline, EMBASE, Psycho INFO, Maternity & Infant Care, LILACS, clinical trial.gov, web of science, SCOPUS, and CENTRAL Database. We also included WHO local databases as follows (see Supplementary Appendix 1 - B).

We checked the references of the reviews and references of the original RCTs to ensure no original study was missed. Our search strategy was designed by our Health Librarian at Tufts, and was approved by WHO counterpart (see Supplementary Appendix 1 - C). We used OpenGrey (https://opengrey.eu), Google, and Google Scholar to obtain relevant grey literature.

The retrieved review articles from the search strategy results were imported into Mendeley to remove duplicate copies and exported to Covidence software (Veritas Health Innovation, Melbourne, Australia). A PRISMA flow diagram was used to show the selection of the studies.

Title and abstract screening was conducted using Covidence and our eligibility criteria. Our team of ten reviewers obtained a Kappa score of more than 7 in pairs. Upon inclusion, the studies were imported into the second stage of screening, a full-text screening for further assessment of their appropriateness. Studies that were approved after full-text review underwent quality appraisal and data extraction. One reviewer extracted the data while the other reviewed the data extraction for accuracy. The second data extractor was responsible for creating forest plots in Revman when feasible. Reasons for exclusion were documented in the PRISMA flow diagram. Covidence identified evaluation differences between reviewers at each stage of the selection process and, subsequently, disagreements were resolved by discussion.

Studies were separated into three major categories: RCTs, cohort, and case control. When we had two or more studies for a comparison and outcome, a forest plot was created. For studies not included in the forest plot, a short summary of the study was placed in Supplementary Appendix 1 - D (5–23).

### Quality assessment

We assessed the risk of bias (high, moderate, or low) in each included study following EPOC criteria (for observational studies) and the Cochrane Handbook for Systematic Reviews of Interventions (24). For each RCT, we examined sequence generation, allocation concealment, blinding, incomplete outcome data, selective outcome reporting, and other potential biases. For quasi-randomized trials, we used the GRADE (Grades of Recommendation, Assessment, Development, and Evaluation) “risk of bias” framework, which is reported in the eligibility criteria, exposure measurement, outcome, confounding, and attrition rates (25).

For observational studies, we used the Dawn and Black quality assessment tool. This tool contains items such as clarity and external and internal validity (bias, exposure, and confounding) and culminates in a numerical score (26, 27).

### Analysis

The unit of analysis was the individual participants in each study. The analysis of this review was limited to the analytical method used in the trial report (e.g., intent to treat, per protocol, or a modification of either type). Studies were combined for meta-analysis only when identical modern contraceptive devices, tools, or drugs; dosages; and regimens could be compared. We calculated the rate ratios (RR) and hazard ratios (HR) with a 95% confidence interval (95% CI) for each dichotomous outcome. We created forest plots for three-time points when possible: less than 5 years, more than 5 years, more than 10 years. If outcomes were reported for multiple reference periods, we reported the outcomes for the first and last reference periods only.

### Subgroup analysis and sensitivity analysis

Subgroup analysis was conducted by comparing different types of contraception, dose, and route of administration when possible. We also conducted a sensitivity analysis to test the robustness of any results that appeared to be based on heterogeneous combinations by examining the effect of deleting each study. Finally, sensitivity analyses were conducted based on rates of loss to follow-up and any study with a rate of loss to follow-up over 20% was excluded.

### Assessment of heterogeneity

We conducted a meta-analysis if two data points or more presented themselves for each comparison and outcome. We visually examined heterogeneity by comparing study designs, target populations, and primary outcome measures across included studies. We assessed the homogeneity of trials combined in a meta-analysis using both fixed-effects and random-effects models. The classical measure of heterogeneity is Cochran's Q, which was calculated as the weighted sum of squared differences between individual study effects and the pooled effect across studies. Q is distributed as a chi-square statistic, and the alpha level is set at 0.10. We then used the I^2^ score to identify the magnitude of heterogeneity. Any score of I^2^ above 50% was investigated for the clinical and methodological diversity of the studies.

GRADE profiler (GRADEpro 2020) was used to import data from Review Manager (Revman) 5.3 to create a “Summary of findings” table. A summary of the intervention effect and a measure of quality for each of the above outcomes was produced using the GRADE approach, which involves five considerations (study limitations, consistency of effect, imprecision, indirectness, and publication bias) to assess the quality of the evidence for each outcome.

## Results

**Figure 1:**
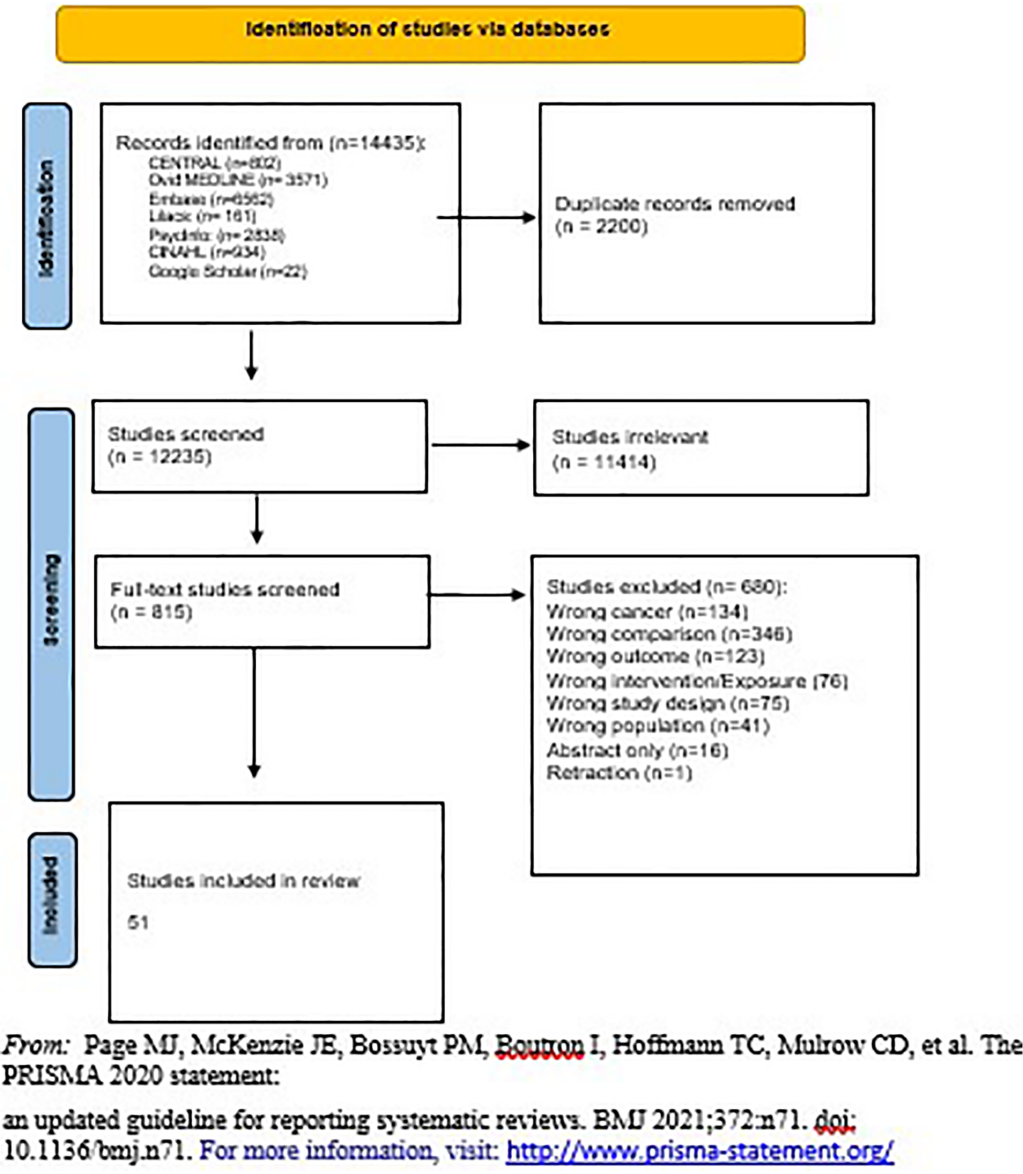
PRISMA flow diagram.

The Prisma chart in Figure 1 demonstrates the number of studies included in the search from different sources as well as the number of studies screened and included in the review. Separate publications discuss the connections between family planning methodologies and other non- reproductive health outcomes including irregular menstruation and changes in mental health.

The total number of included studies was 51, 2 of which were randomized clinical trials (RCTs) (7, 8) and 49 of which were observational studies (7 case-control studies (14, 15, 28–32) and 42 cohort studies (5, 6, 9–13, 16–23, 33–60). Supplementary Appendix 2 - Table S1 shows characteristics of the RCTs, including country of origin, year of publication, number of facilities, type of health facility, level of health facility, sample size, study design, population, type of contraception studied, the outcome of interest extracted, and quality of study based on design. Similar data (with exposure instead of intervention) was extracted for observational studies (Supplementary Appendix 2 - Table S2). Most of the studies were from 2000 onward, while eight were published before 2000.

Studies focused on either one form of contraception (OCPs, ring/patch, implant, injection, IUD, condoms, sterilization), a combination of contraceptives, or all HCs.

Comparisons were set based on available literature and the protocol on either all HCs vs. no contraceptive use or OC use vs. non-use. In cases where other types of contraceptives were studied, comparisons were made between use and non-use. Subgroup analysis can be seen is some of the forest plots where different contraceptives are used (e.g., OCPs vs. IUDs).

Outcomes of interest were morbidities and mortalities related to and reproductive cancers.

### Quality assessment

**Figure 2:**
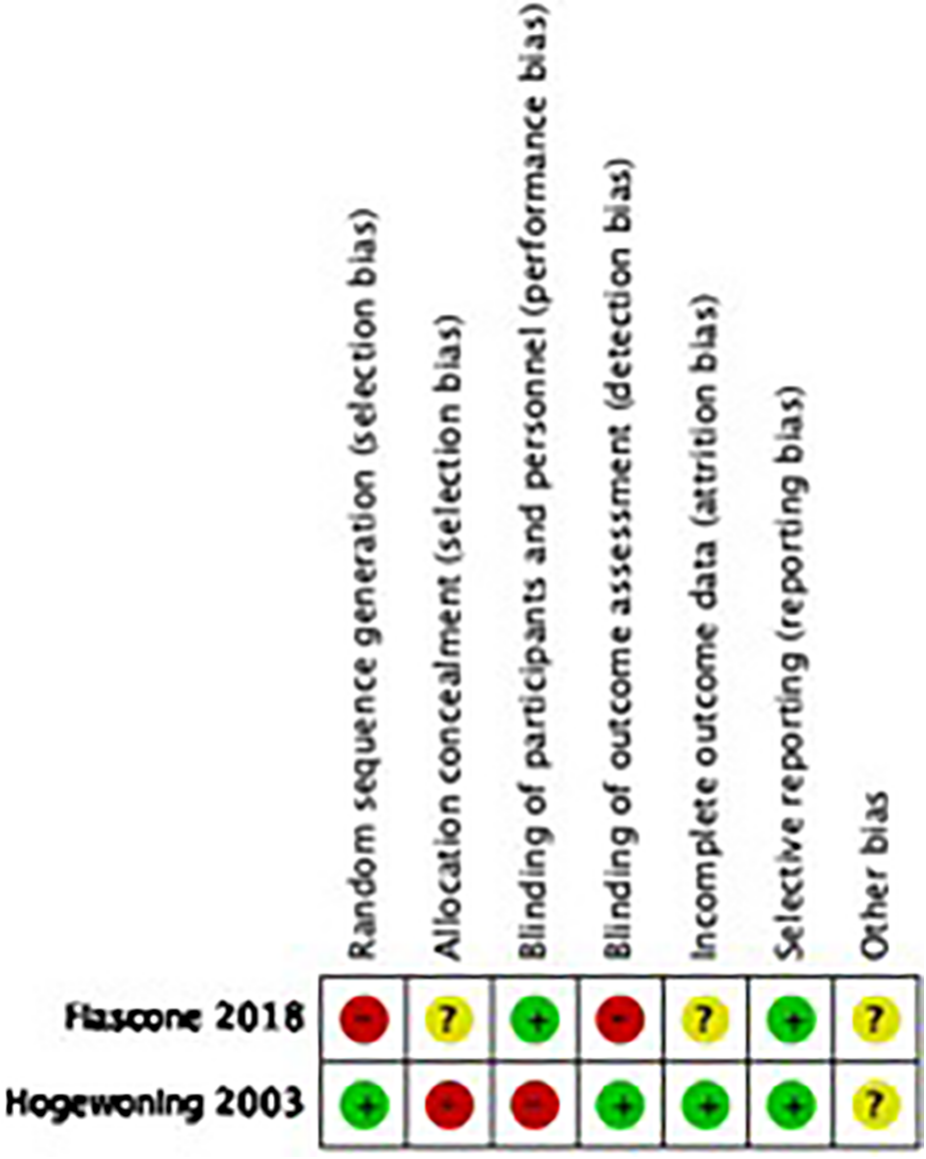
Risk of bias graph for RCT included studies.

Figure 2 reflects the quality assessment of included studies and is presented below.

Supplementary Appendix 2 -Table S3 shows the quality assessment of observational studies using Dawn and Black scoring system. We considered the overall quality of evidence to be moderate for our review (mean: 13.24 ± 2.25, median = 14 min = 5, max = 16).

### Cohort studies

#### Ovarian cancer

We found 8 cohort studies that investigated OC use in relation to ovarian cancer rate (33–36, 39–41, 57), three investigated hormonal IUDs (36, 43, 46), two studies analyzed injectable contraception (36, 44), and three studies compared any hormonal contraceptive users to non- users (36, 39, 45).

**Figure 3:**
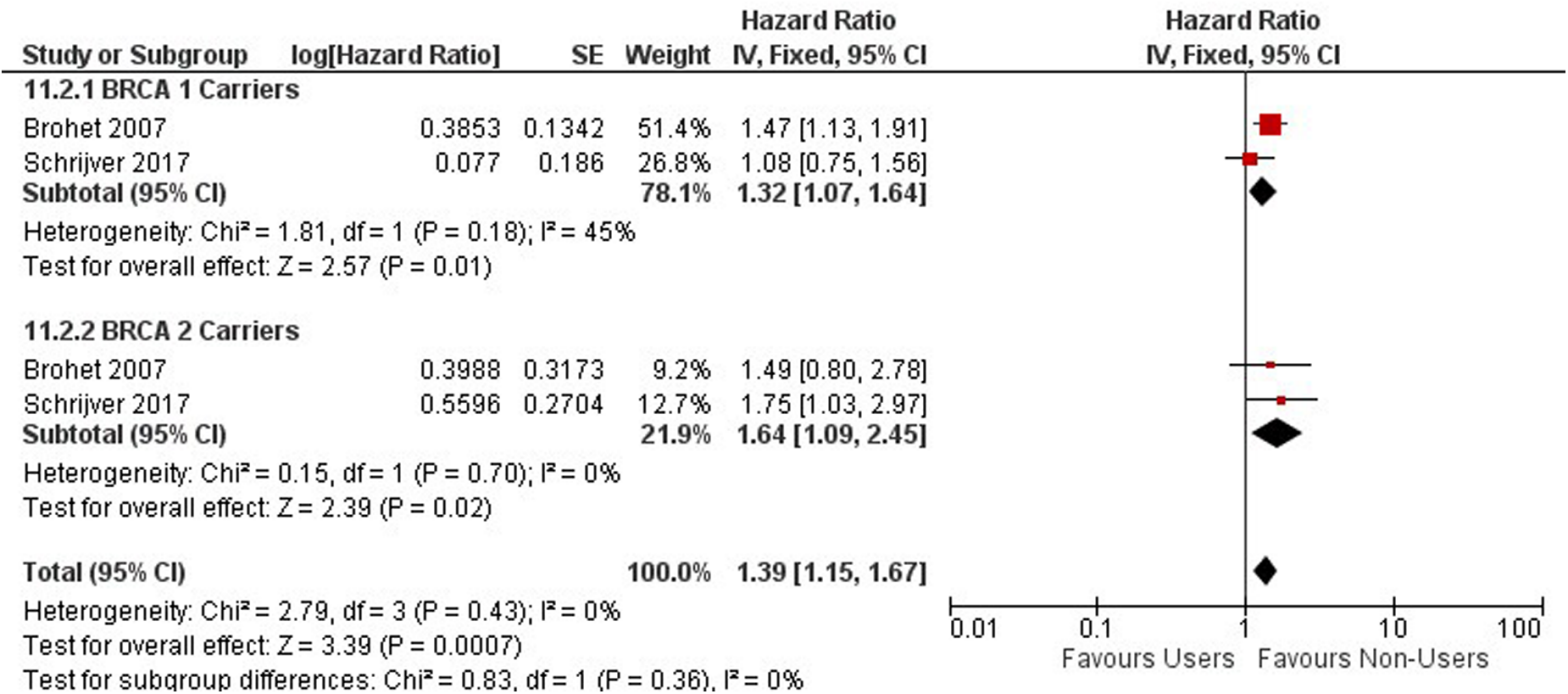
Forest plot on hormonal contraceptive methods and ovarian cancer.

Compared with non-users, all hormonal contraceptive users had a 36% lower rate ratio of ovarian cancer (0.64, 95% CI 0.60, 0.68). We stratified the type of contraceptive use into OCP (0.62, 95% CI 0.57–0.68), hormonal IUS (0.68, 95% CI 0.48, 0.96), injectables (1.34, 95% CI 0.81, 2.21), and any contraceptives (oral or non-oral) (0.64, 95% CI 0.58, 0.71) based on what studies presented (Figure 3).

Five studies provided information on the hormonal contraception dosing regimens used among women and compared them to non-hormonal method users (35, 36, 39, 43, 44). Progestogen-only products include oral (Norethisterone, Levonorgestrel, Desogestrel) and non-oral (MPA depot, Implant, LNG-IUS) methods (36). Combined HC includes oral ethinylestradiol and non-oral patch and vaginal ring. (Supplementary Appendix 3-Figure S1) Norethisterone 50 mg ethinylestradiol, Levonorgestrel 50 ethinylestradiol, Norethisterone 30–35 mg ethinylestradiol, Drospirenone 20–35 mg ethinylestradiol, Noregestimate 35 mg ethinylestradiol, Norethisterone Progestin- Only Oral, Levonorgestrel Progestogen-Only Oral, Hydroxyprogesterone Caproate 5 mg Estradiol Valerate, and Progestin only methods (oral and non-oral) showed no significant effect on ovarian cancer risk. The following regimens were significantly associated with a reduced risk in ovarian cancer incidence compared to non-users: Levonorgestrel 30–35 mg ethinylestradiol (RR 0.33, 95% CI 0.18–0.61), Desogestrel 20–30 mg ethinylestradiol (RR 0.45, 95% CI 0.27–0.75), Gestodene 20–35 mg ethinylestradiol (RR 0.57, 95% CI 0.41–0.79), LNG-IUS Progestogen (RR 0.68, 95% CI 0.48–0.96), and any combined oral pills (RR 0.56, 95% CI 0.49–0.64). Over a 6-fold increase in ovarian cancer incidence was observed among women using MPA Depot Progestogen compared to non-users of hormonal contraception (RR 6.56, 95% CI 2.11–20.39).

**Figure 4:**
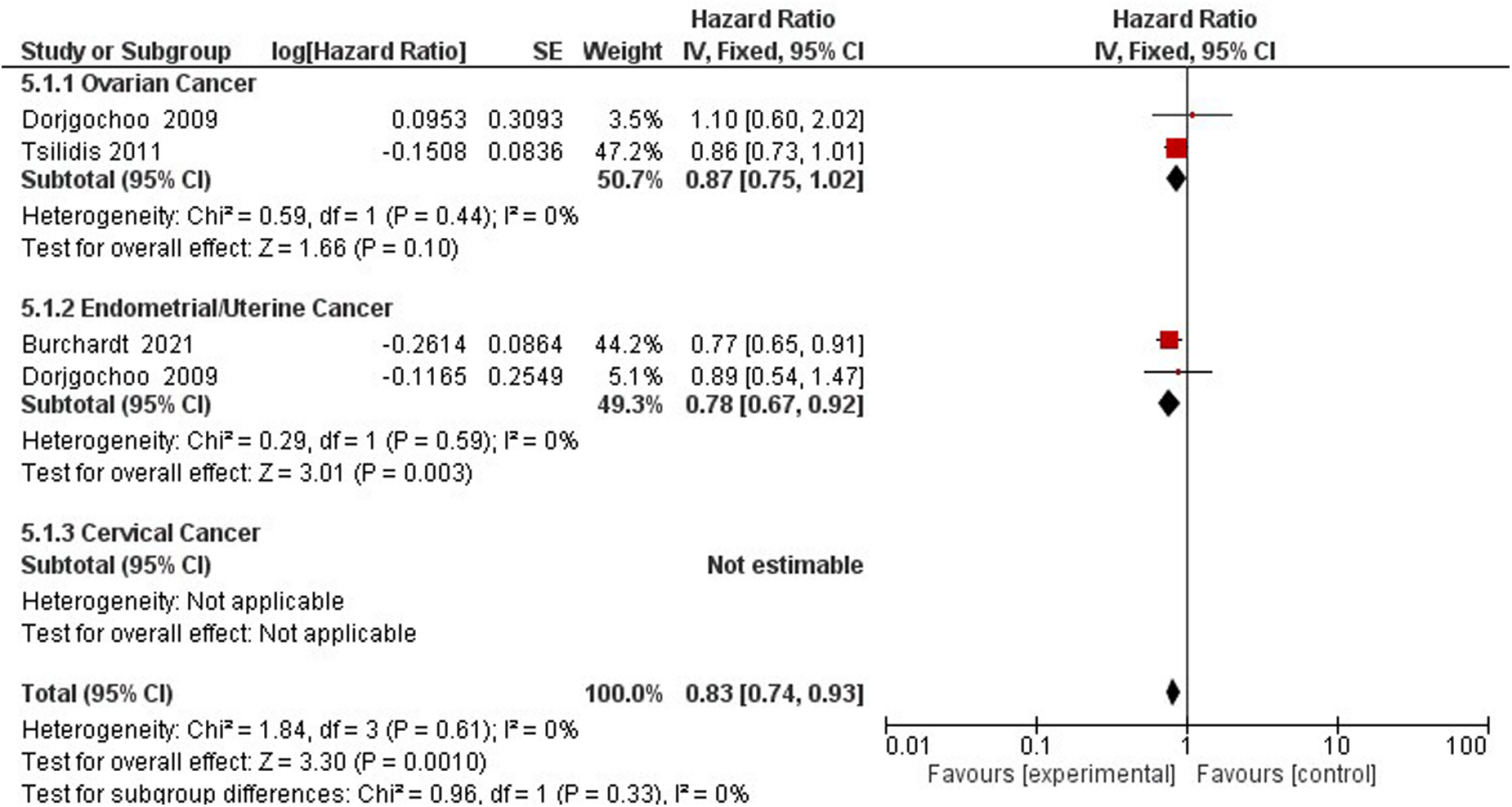
Forest plot on No-hormonal contraceptive methods and ovarian cancer.

One study with 2,479,493 person-years from 107,900 women and 861 confirmed ovarian cancer cases (1,243 cases self-identified), investigated nonhormonal methods of contraception on the relative risk of ovarian cancer (46). Compared with non-users, there was no increased or decreased risk of ovarian cancer among non-hormonal method users (RR 0.92; 95% CI 0.80–1.06) (see Figure 4). We stratified the type of non-hormonal contraceptive methods and found significant protective effects for tubal ligation (RR 0.66; 95% CI 0.50–0.87). Conversely, we identified an increased risk by 76% in women using copper IUD's (RR 1.76; 95% CI 1.08–2.87).

### Endometrial cancer

**Figure 5:**
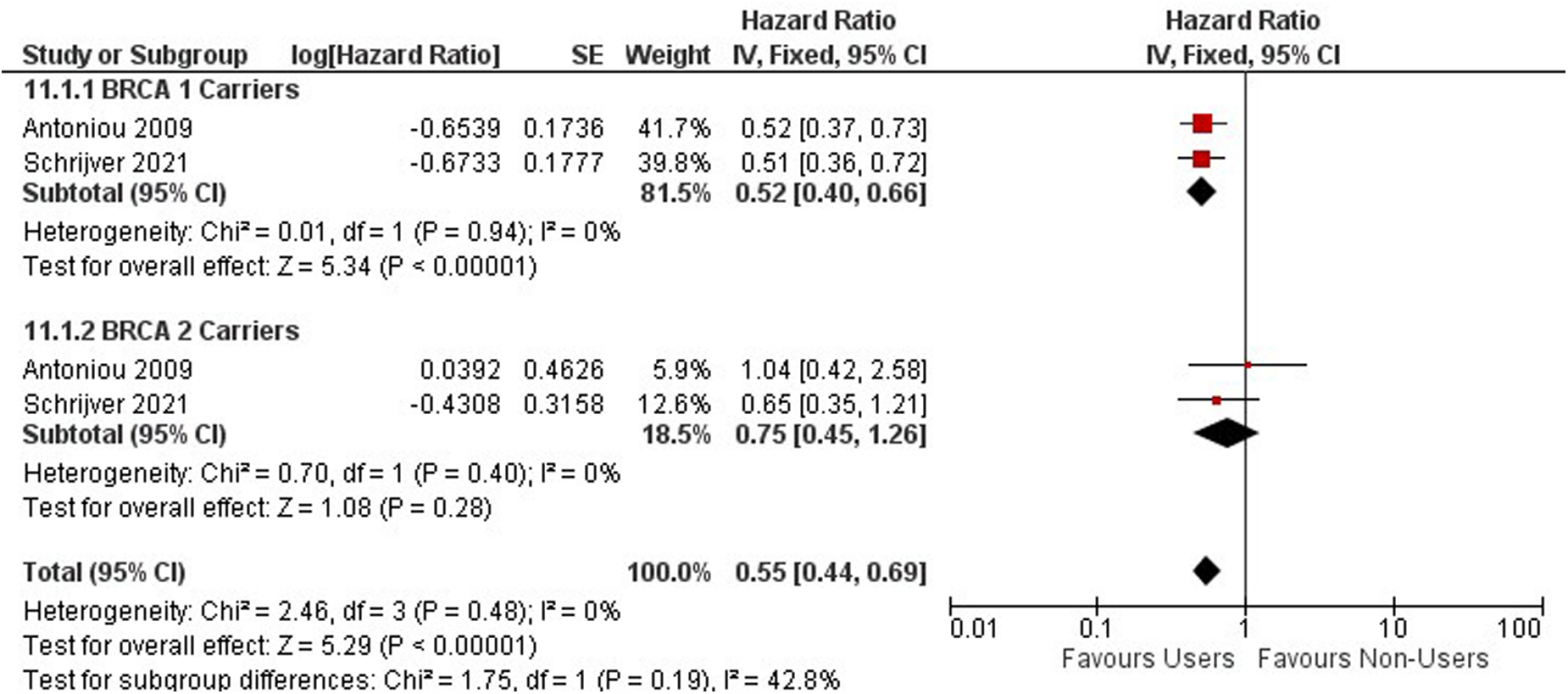
Forest plot on hormonal contraceptive Use and endometrail cancer.

Contraceptive users had a significantly lower risk of endometrial cancer incidence when compared to non-users (0.56, 95% CI 0.50, 0.63). We identified six studies on oral contraceptives (33, 35, 37, 40, 41, 57), two studies on hormonal IUS (36), one study on progestin-only products (37), and one study for any contraceptives (oral and non-oral) (37). The rate ratio for oral contraceptive users was 0.53 (95% CI 0.45, 0.63), for hormonal IUS 0.58 (95% CI 0.42, 0.79), for progestin-only products 0.73 (95% CI 0.23, 2.32) and for any contraception 0.61 (95% CI 0.49, 0.76). All hormonal contraception methods had a significant downward trend for endometrial incidence, except progestin-only products had no significant results (Figure 5).

Three studies investigated the associated risk incidence of endometrial cancer by comparing varying dosing regimens to non-users (37, 43, 44).

A significant protective effect against endometrial cancer incidence was observed among the following dosing regimens: Gestodene 20–35 mg ethinylestradiol (RR 0.27, 95% CI 0.12–0.61), LNG-IUS (Supplementary Appendix 3 - Figure S2).

Progestogen Only Non-oral (RR 0.22, 95% CI 0.13–0.37), Alpha-hydroxyprogesterone Caproate and 5 mg Estradiol Valerate (RR 0.27, 95% CI 0.12–0.61), and any combined oral pills (RR 0.66, 95% CI 0.48–0.91). A different cohort with 116, 429 females observed protective effects of estrogen or progestin formulations against endometrial cancer (HR 0.77; 95% CI 0.69–0.85) (60) (Figure 8).

### Cervical cancer

**Figure 6:**
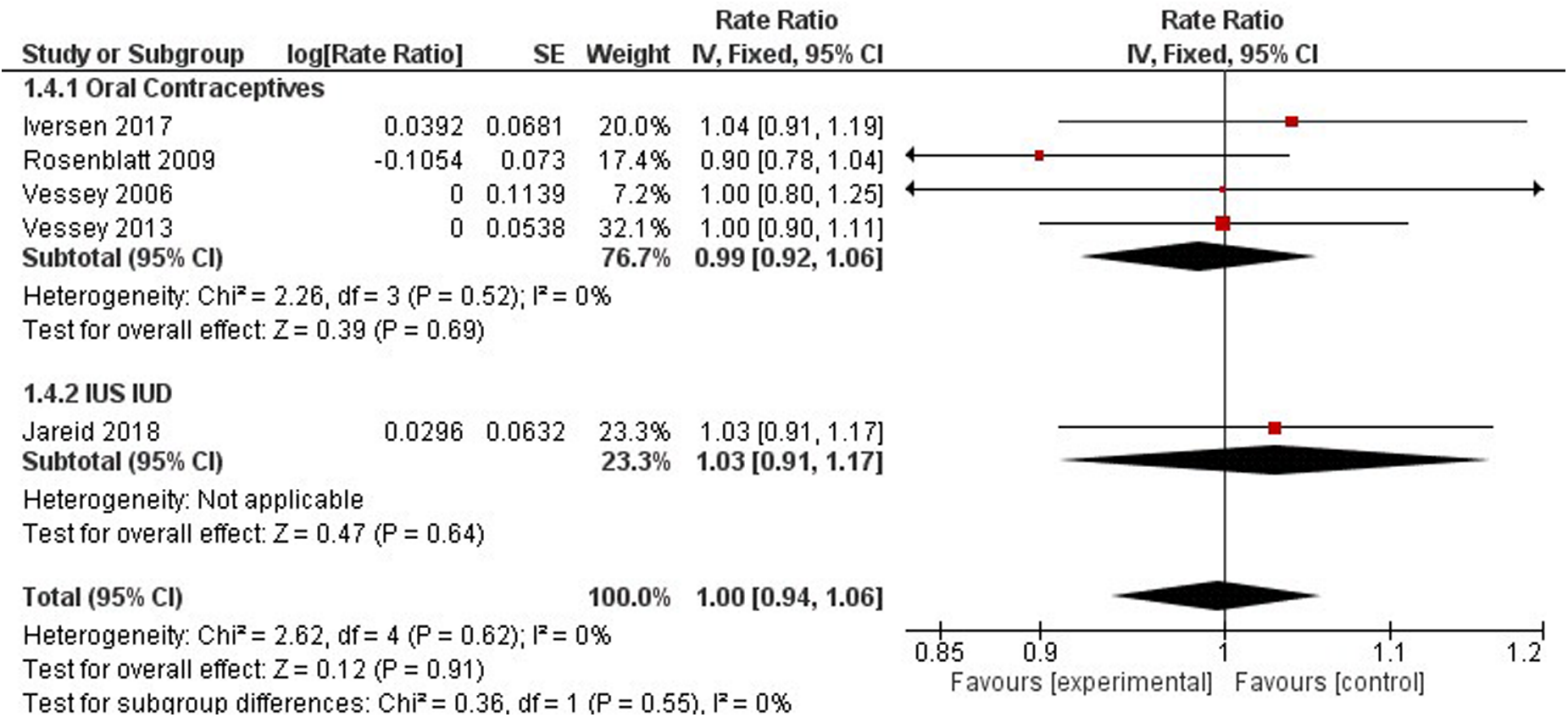
Forest plot on hormonal contraceptive Use and cervical cancer.

**Figure 7:**
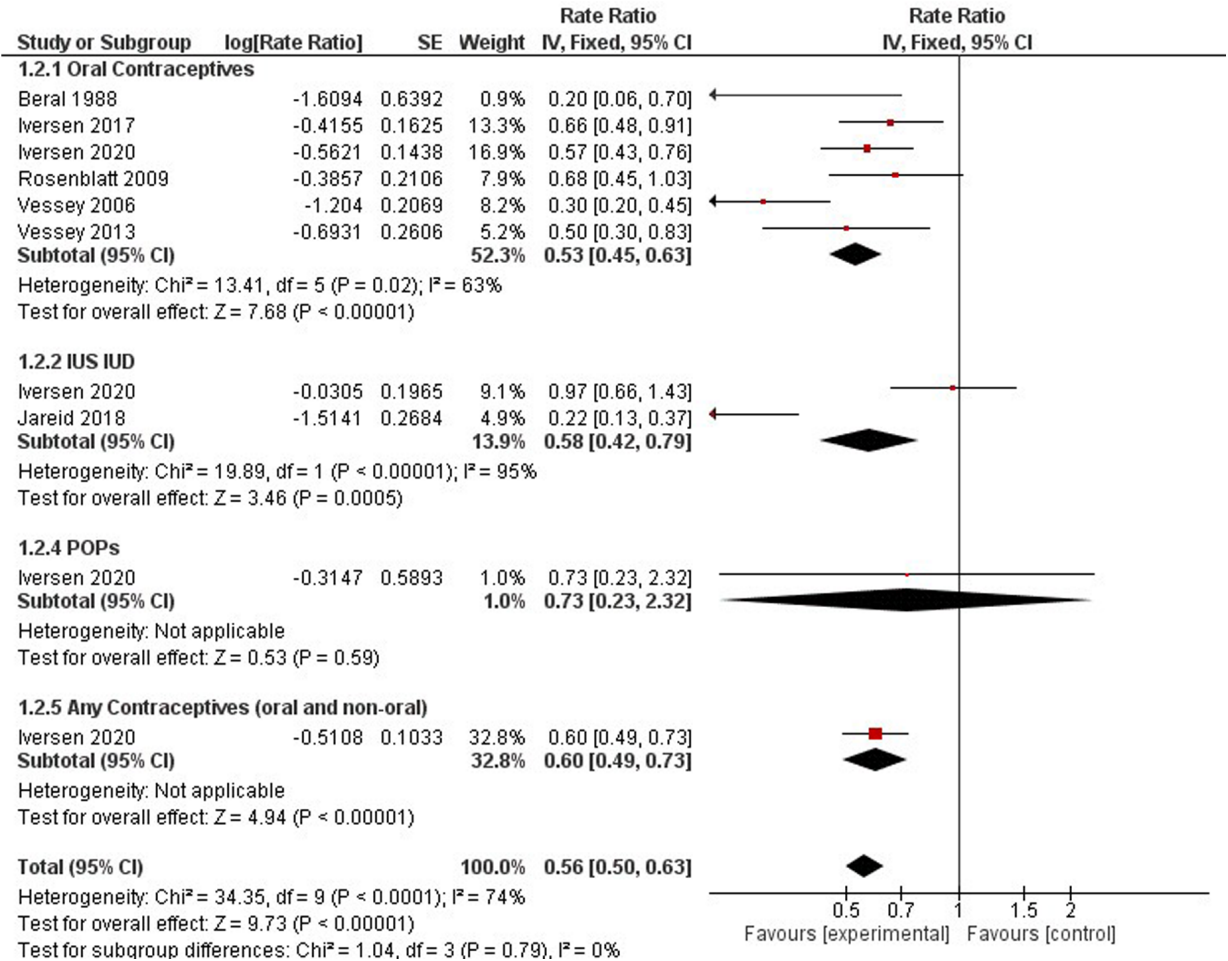
Forest plot on OCP use and gynecological cancer hazard.

Cervical cancer was studied by six studies on oral contraceptives (33, 35, 38, 40, 41, 57), one study on hormonal IUS (38), and one study for any contraceptives (oral and non-oral) (38) (Figure 7). The rate ratio of cervical cancer was higher among non- users compared to hormonal contraceptive users when we pooled the results (1.28, 95% CI 1.21, 1.35). When stratifying the results, a significant increased risk was observed for OCP users (RR = 1.43, 95% CI 1.31, 1.56) and any users of oral and non-oral (RR = 1.19, 95% CI 1.10–1.29), while women using IUS showed no significant effect on cervical cancer risk incidence (RR = 0.76, 95% CI 0.52–1.11). In a comparison between non-users and hormonal IUD users, the risk ratio was 0.58(0.42, 0.79). Any contraceptive users reported one study only (37). These results had high heterogeneity (*I*^2^ = 80%) (Figure 6).

When comparing dosing regimens, a nonsignificant downward trend in cervical cancer incidence was observed by Rosenblatt et al., 2007 among Hydroxyprogesterone Caproate and 5 mg Estradiol Valerate users (RR 0.54, 95% CI 0.07–4.17), while an upward trend was observed by Iversen et al., 2017 for any combined oral contraceptive users (RR 1.31, 95% CI 0.82–1.94).

### Gynecological cancer

The association between gynecological cancer and the protective effect of tubal ligation was reported by four studies (47–50). Tubal ligation reduces the hazards of gynecological cancer risk by 20% (HR = 0.80, 95% CI 0.66–0.87) (Supplementary Appendix 3 - Figure S3).

**Figure 8:**
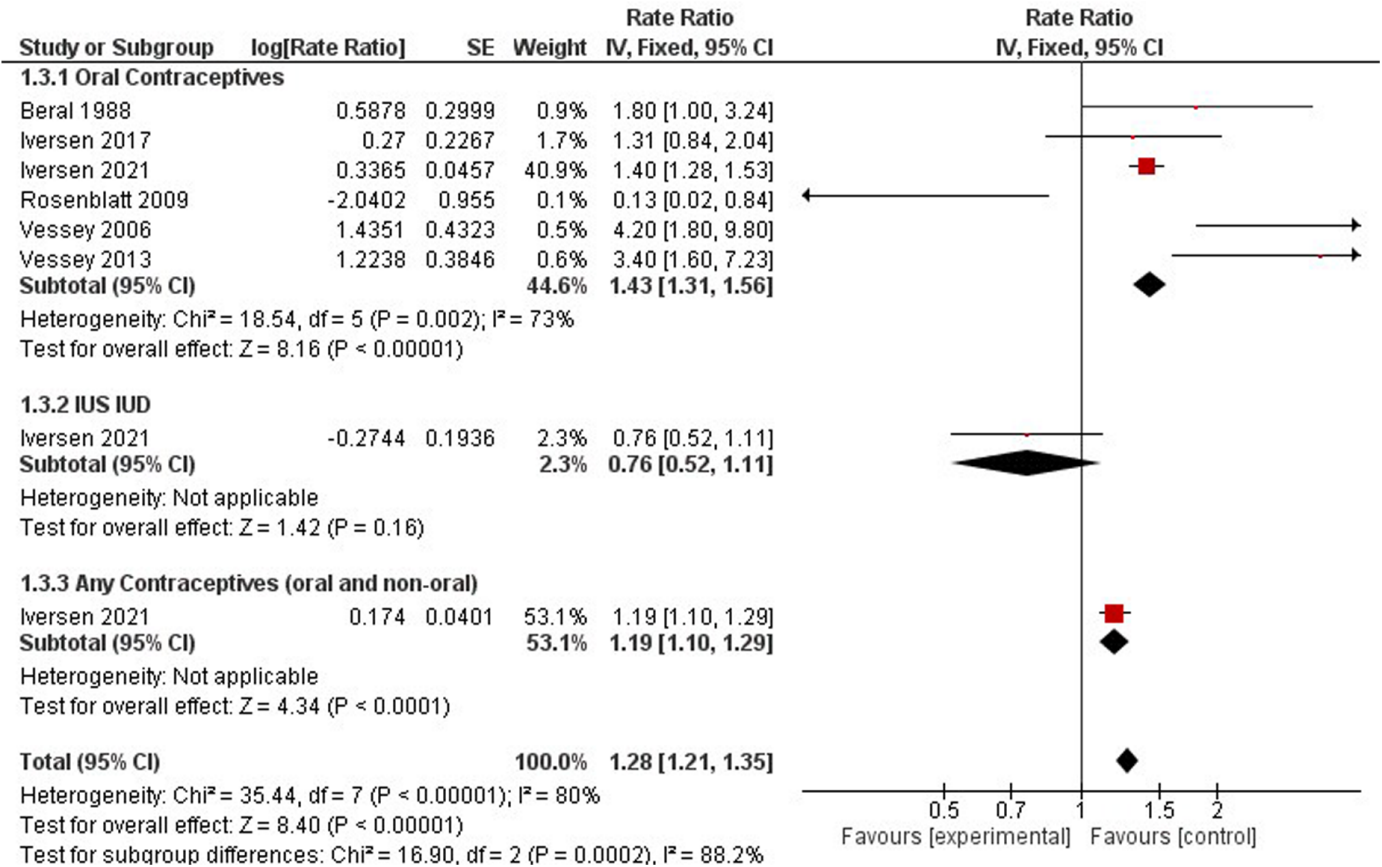
Forest plot on hormonal contraceptive Use and breast cancer.

Three studies assess the hazards of gynecological cancer among OCP users and non-users (22, 47, 60) (Figure 7). Compared to non-users, OCP use reduces the hazard of gynecological cancers of the ovaries and endometrium by 17% (HR 0.83; 95% CI 0.74–0.93) (Figure 8).

Four studies assessed the association between hormonal contraception usage and cancer mortality (42, 51–53). Compared to non-users, a reduced incidence of gynecological cancer mortality was observed among HC users (RR 0.60, 95% CI 0.51–0.71). When stratifying the results by gynecological cancer type, a 63% increase in cervical cancer mortality was observed among HC users (RR 1.63, 95% CI 1.07–2.49). Similarly, a cohort study containing 121,577 participants found a slight protective effect when comparing ever users of OCs to never users (HR 0.87; 95% CI 0.77–0.98) (59) (Supplementary Appendix 3 - Figure S4).

### Breast cancer

Breast cancer was studied by four studies on oral contraceptives (35, 41, 57), and one study on hormonal IUS (43) (Figure 8). A pooled non-significant rate ratio was estimated (1.00, 95% CI 0.94, 1.06) for OCP and IUS users. Dosing regimens comparing HC users to non-users found no significant difference in breast cancer incidence among Hydroxyprogesterone Caproate and 5 mg Estradiol Valerate, LNG-IUS Progestogen-Only Non-Oral, and any Combined Oral Pill users with a pooled effect of 1.02 (95% CI 0.93–1.11) (35, 43, 44) (Figure 8).

Only one study observed the association between tubal ligation and breast cancer risk (47). No significant effect was observed among women who underwent tubal ligation and those who have not (RR 1.15, 95% CI 0.86–1.54). Three studies investigated regimen dosing to understand breast cancer risk among HC users (35, 43, 44). Compared to non-users, no significant difference in breast cancer incidence was observed among women using alpha- hydroxyprogesterone caproate and 5 mg estradiol valerate, LNG-IUS Progestogen-Only Non- Oral, and any combined oral pills (RR 1.02, 95% CI 0.93–1.11).

Additionally, breast cancer mortality was not significantly different between OCP users and non-users (1.00, 95% CI 0.89, 1.12) (Supplementary Appendix 3 - Figure S5).

### Contraceptives and hereditary cancers Ovarian cancer

**Figure 9:**
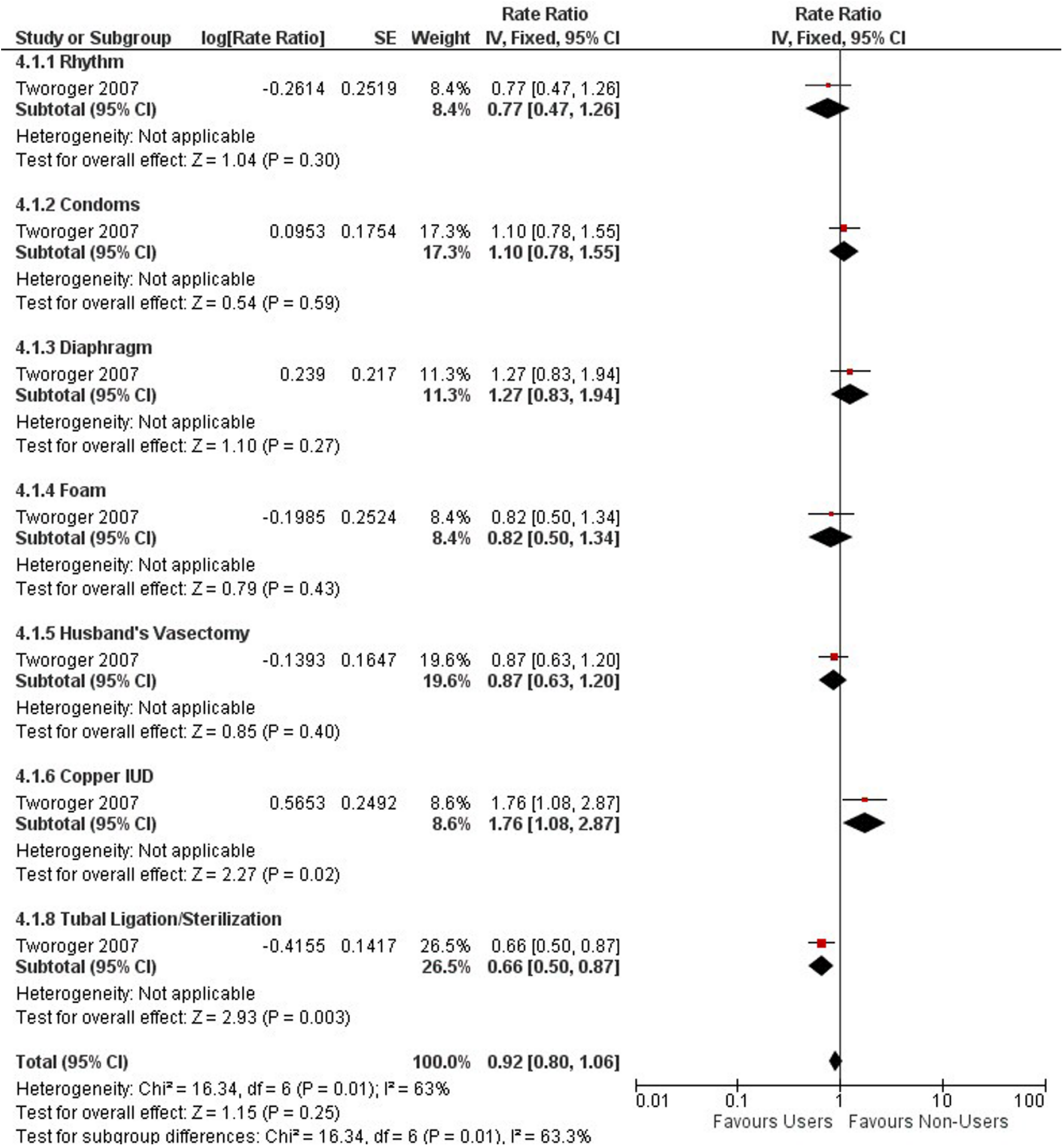
Forest plot on oral contraceptive Use and ovarian cancer by mutation carries.

Ovarian cancer risk among BRCA 1 and 2 mutation carriers using OCs was investigated within two cohort studies including 9,753 women (54, 58) (Figure 9). Among BRCA 1 and 2 mutation carriers, ever users of oral contraceptives are associated with a decreased risk of ovarian cancer (HR = 0.55, 95% CI 0.44, 0.69) (Figure 9).

Among BRCA 1 and 2 carriers using OCs for less than 5 years, no significant effect was observed (HR = 0.76; 95% CI 0.57, 1.02) Supplementary Appendix 3 - Figure S6.

A significant protective effect against ovarian cancer is observed among BRCA 1 and 2 Carriers who use oral contraceptives between 4 and 9 years (HR = 0.45; 95% CI 0.34, 0.61) (Supplementary Appendix 3 - Figure S7).

For BRCA 1 carriers, OCPs have a protective effect, while no difference in effect is detected among BRCA 2 carriers. BRCA 1 and 2 mutation carriers using oral contraceptives for greater than 10 years have a significant protective effect against the risk of ovarian cancer (HR = 0.37; 95% CI 0.26, 0.54). (Supplementary Appendix 3 - Figure S8).

### Breast cancer

**Figure 10:**
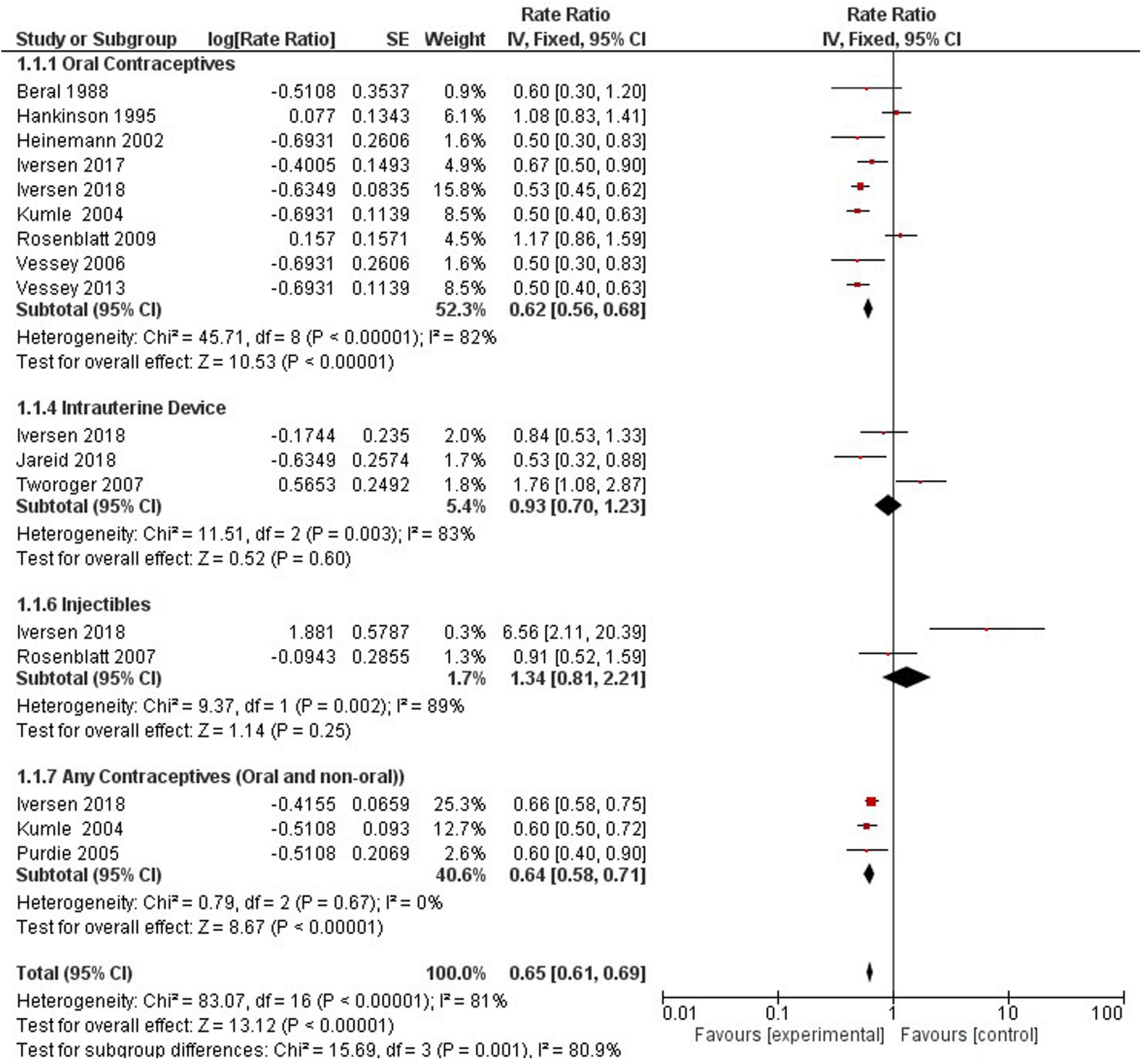
Forest plot on oral contraceptive Use and breast cancer by mutation carries.

The risk of breast cancer association from OC use was observed among 11,432 BRCA 1 or 2 gene mutation carriers with two cohort studies conducted in the United Kingdom and Netherlands (55, 56) (Figure 10). Among BRCA 1 and 2 mutation carriers, ever users of oral contraceptives are associated with an increased risk of breast cancer compared to never users (HR = 1.39, 95% CI 1.15, 1.67).

Among BRCA 1 and 2 carriers using OCs for less than 5 years, an increased risk in breast cancer was observed compared to never users (HR = 1.33; 95% CI 1.07, 1.66) (Supplementary Appendix 3 - Figure S9).

A significant increase in risk of breast cancer is observed among BRCA 1 and 2 Carriers who use oral contraceptives between 4 and 9 years compared to never users (HR = 1.29; 95% CI 1.03, 1.61), however, the risk between carriers using OCs for less than 5 years and between 4 and 9 years are very similar as you compare them (Supplementary Appendix 3 - Figure S10).

BRCA 1 and 2 mutation carriers using oral contraceptives for greater than 10 years have an increased risk of breast cancer (HR = 1.47; 95% CI 1.18, 1.85). Long term OC use of greater than 10 years has a higher risk for mutation carriers as compared to those using them for less than 10 years (Supplementary Appendix 3 - Figure S11).

### Duration gynecological cancers

Duration of hormonal contraception use among a sample of over 21 million women were accounted for in 6 cohort studies measuring the rate ratio of gynecological cancers among users and non-users to examine a dose-response relationship (10, 37, 39–41, 57) (Supplementary Appendix 3 - Figures S12, S13).

Among short-term hormonal contraception users who used HCs for less than 5 years, a significant protective effect was observed for ovarian (RR = 0.63; 95% CI 0.52, 0.75) and endometrial cancer (RR = 0.75; 95% CI 0.59, 0.94) outcomes, however, a harmful effect was observed for cervical cancer (RR = 2.55; 95% CI 1.17, 5.59) (Supplementary Appendix 3 - Figure S12).

Among long-term HC users who used HCs for at least 8 years or more, a similar pattern to short-term users was observed. A significant reduction in ovarian (RR = 0.36; 95% CI 0.27, 0.49) and endometrial cancers (RR = 0.26; 95% CI 0.19, 0.36) was observed. A harmful effect was observed for cervical cancer, and this risk increased from the initial risk that short-term users have (RR = 2.55 vs. 5.40; 95% CI 2.89, 10.89) (Supplementary Appendix 3 - Figure S13).

### Breast cancer

There were three cohort studies with 275,988 participants examined for the association between duration of HC use and breast cancer incidence (Supplementary Appendix 3 - Figures S14, S15) (40, 41, 57). No significant relationship among short-term and long-term HC users was observed for breast cancer incidence.

## Discussion

### Summary of main results

There are significant reductions in the rate of ovarian and endometrial cancer incidence and mortality among contraceptive users. Short- and long-term methods showed a protective effect against ovarian and endometrial cancers. When comparing dosing regimens, HCs containing both progestins and estrogens have protective effects against ovarian and endometrial cancer. Conversely, HC users are at an increased risk of cervical cancer incidence and mortality, and this risk persists regardless of duration. Among healthy women with no genetic mutations, no significant difference in breast cancer incidence and mortality was reported among users and non-users of HC. Among mutation carriers (BRCA1/2) using OCs, an increased risk in breast cancer incidence was observed and this risk persists among short- and long-term users. For ovarian and cervical cancer, our case-control findings matched our cohort studies, however, for breast cancer, a significant risk was observed among OCP users.

Contraceptive use as a primary exposure for our review was self-reported in a majority of our studies. Recall bias and information bias are a major concern for studies that relied on self- reports of contraceptive use as this can potentially underestimate the true effect of contraceptive use on our outcomes of interest. Future studies should focus on techniques such as recording the on-time injection checklist or electronic pill count.

Highlighting the mechanism of action in each scenario outlined above is crucial. For example, the inhibition of ovulation is a key factor in providing protection against ovarian cancer, shedding light on why specific methods like DMPA may not confer the same level of protection as COCs. Nevertheless, it's important to note that this manuscript, while acknowledging the importance of theoretical mechanisms, refrains from delving extensively into the intricacies beyond the causality of the effects on different types of cancer.

### Agreements and disagreements with other studies

Our results on contraceptives as a protective effect against ovarian cancer are confirmatory to the initial findings of Havrilesky 2013 (61). A previous study (Pragout et al.) reported an insignificant increased risk of cervical cancer, while our study found a significantly (28%) higher risk among users than non-users (62). In the systematic review conducted by Asthana, the overall odds ratio of invasive cancer on OC use was estimated as 1.51 (95% CI 1.35, 1.68) with 19 included studies, which is not very different from our study (63). In Pragout et al., a slight but significant increase in breast cancer risk among users was observed; however, in our study, no significant differences were observed between users and non-users, except for women who are BRCA1/2 carriers. A study (Conz et al.) systematically reviewed the relationship between use of the Levonorgestrel-releasing intrauterine system (LNG-IUS) and breast cancer risk with 26 studies. They found an increased risk in LNG-IUS users (pooled OR = 1.16, 95% CI 1.06–1.28) (64). However, we did not detect a significant risk in LNG-IUS users. Our study supports the previous findings from Pragout 2018 on the significant reduction of endometrial cancer. However, there is conflicting information about the roles IUD and tubal ligation play in the risk factor. For BRCA1/2 mutation carriers, Huber examined OC users’ risk of ovarian cancer and breast cancer compared to non-users in a systematic review (65). It reported a risk reduction in ovarian cancer, and a significant risk elevation in breast cancer, which is aligned with our findings.

Our study contributes nuanced insights into the complex relationship between contraceptive use and various types of cancer, offering both confirmatory evidence and novel findings that warrant further exploration and validation in future research endeavors.

Our disagreement with other studies can also be explained by the adopted methodology that we employed a comprehensive approach by searching across 19 databases to ensure inclusivity. Papers were included without geographical or language restrictions. The population of interest encompassed women of reproductive age (14–49 years). Interventional studies were included if they investigated modern contraceptive methods endorsed by the WHO, such as short-acting hormonal contraception, long-term contraception, one-time barrier contraception, permanent contraception, and emergency contraception. Observational studies considered contraceptive use of all types as the main exposure, excluding those investigating contraception alongside other medications or modalities. Comparison groups consisted solely of non-users of contraception. The primary outcome of interest was mortality and morbidity related to reproductive tract cancers. Eligible study designs included parallel, or cluster randomized controlled trials, controlled clinical trials, controlled before and after studies, interrupted time series studies, cohort or longitudinal analyses, regression discontinuity designs, and case-control studies.

### Strengths

First, we searched the databases for all studies from their inception. Thus, we included studies with both outdated and up-to-date contraceptive methods, with broad coverage. Second, for each outcome category, our studies covered diseases and conditions commonly seen in women of reproductive age. For some of these outcomes, we have studies that yielded large pooled sample sizes and low loss to follow-up rates, allowing for higher statistical power, narrower confidence intervals, and more credible results. The prospective study design eliminated the possible influence of differential recall of contraception use.

### Limitations

Cancer is a difficult outcome to measure as there is a long latency period between the time of exposure and the detection of an outcome. During these long latency periods, the methods and dosing regimens a woman uses can change over time. Our study did not account for the age at first use of contraceptives, time since last use, environmental exposures, or demographic characteristics. Included studies lacked information on specific HC regimens and dosing and HC methods (patch/ring, implants, and injectables) were often left out of analysis due to a lack of available data. There were limited studies examining the association between varying HC methods and dosing regimens for cervical and breast cancer outcomes. Most studies were on OC users, with limited studies looking at the association of long-term contraceptive types. As long-term contraceptive methods become more popular, it is essential to have more information on these methods. Heterogeneity was a significant issue in this study.

### New knowledge this review brings to the field

This review provides new insights into the relationship between modern contraceptive use and cancer risk, focusing on the effects of birth control pills, and long-acting reversible contraceptives. We found a significant reduction in the risk of developing ovarian and endometrial cancers among contraceptive users, highlighting the potential benefits of these contraceptive methods. However, the review also reveals an increased risk of cervical cancer among contraceptive users, which persists regardless of the duration of use. These findings suggest the need for additional research into the link between hormonal contraceptive use and cancer incidence. The authors recommend that healthcare providers consider various factors such as age, physical and mental status, family history, and individual preferences when selecting contraceptive methods to maximize benefits and minimize risks.

### Implications for practice

The findings of this research offer a comprehensive body of evidence on the utilization of contraception and its impact on various stakeholders, such as healthcare professionals, pharmaceutical companies, pharmacists, and others. The results indicate that aside from its primary role as a contraceptive, contraception may also have preventive or therapeutic properties for certain diseases or conditions. Nevertheless, the study highlights potential adverse effects that must be considered. Healthcare providers should evaluate several situational factors, such as age, physical and mental health status, and family history, to choose the most suitable contraceptive method for their female patients, ensuring maximum benefits and minimal risks. Recommendations include discussions about the woman's overall health and changes to medical history, continued monitoring and a complete physical examination at each follow-up visit checking for symptoms of blood pressure changes, weight changes, headaches, or abnormal vaginal bleeding (66).

## Conclusion

This comprehensive review explores the intricate relationship between contraceptive use and cancer, revealing significant reductions in ovarian and endometrial cancer incidence and mortality among users. Short- and long-term methods exhibit protective effects against these cancers, especially hormonal contraceptives containing both progestins and estrogens. However, an increased risk of cervical cancer is observed among contraceptive users, persisting regardless of use duration. No significant differences in breast cancer risk were found for healthy women, but for BRCA1/2 carriers using oral contraceptives, an elevated risk was noted. The study's strengths lie in exhaustive database searches, broad coverage of diseases, and a prospective design, minimizing recall bias. Yet, limitations include the long latency of cancer, changing contraceptive methods, and limited information on specific regimens. Overall, this research offers nuanced insights and implications for healthcare practices, suggesting contraception's potential preventive or therapeutic properties for certain diseases. Future research should explore the benefits of different hormonal methods and doses, considering non- reproductive health and specific health conditions in study populations.

## Data Availability

The original contributions presented in the study are included in the article/Supplementary Material, further inquiries can be directed to the corresponding author.
