# Corrigendum: Assessing the impact of contraceptive use on reproductive cancer risk among women of reproductive age—a systematic review

**DOI:** 10.3389/fgwh.2024.1530251

**Published:** 2024-12-09

**Authors:** Shayesteh Jahanfar, Julie Mortazavi, Amy Lapidow, Cassandra Cu, Jude Al Abosy, Kathyrn Morris, Juan Camilo Becerra-Mateus, Meredith Steinfeldt, Olivia Maurer, Jiang Bohang, Paola Andrenacci, Marwa Badawy, Moazzam Ali

**Affiliations:** ^1^ Department of Public Health and Community Medicine, Tufts University School of Medicine, Boston, MA, United States; ^2^ School of Medicine, Tufts University School of Medicine, Boston, MA, United States; ^3^ Department of Public Health and Community Medicine, Universidad de Antioquia, Medellin, Columbia; ^4^ Cochrane US Mentoring Program, Boston, MA, United States; ^5^ US Mentoring Program, Boston, MA, United States; ^6^ WHO Department of Sexual and Reproductive Health and Research, World Health Organization, Geneva, Switzerland

**Keywords:** contraceptives, ovarian cancer, cervical cancer, endometrial cancer, breast cancer

A Corrigendum on Assessing the impact of contraceptive use on reproductive cancer risk among women of reproductive age—a systematic review By Jahanfar S, Mortazavi J, Lapidow A, Cu C, Al Abosy J, Morris K, Becerra-Mateus JC, Steinfeldt M, Maurer O, Bohang J, Andrenacci P, Badawy M, Ali M (2024). Front. Glob. Womens Health. 5:1487820. doi: 10.3389/fgwh.2024.1487820

**Error in Figure/Table**

In the published article, there was an error in the quality of the Figures is poor quality for 3, 4, 5, 6, 7, 8, 9, 10 as published. It is difficult to read the figures.

So I am resending figures in high quality in two formats of: PDF and PNG.

**The figures are as under:**

FIGURE 3 Forest plot on hormonal contraceptive methods and ovarian cancer.

FIGURE 4 Forest plot on No-hormonal contraceptive methods and ovarian cancer.

FIGURE 5 Forest plot on hormonal contraceptive Use and endomentrail (Correct spelling is endometrial) cancer.

FIGURE 6 Forest plot on hormonal contraceptive Use and cervical cancer.

FIGURE 7 Forest plot on OCP use and gynecological cancer hazard.

FIGURE 8 Forest plot on hormonal contraceptive Use and breast cancer.

FIGURE 9 Forest plot on oral contraceptive Use and ovarian cancer by mutation carries.

FIGURE 10 Forest plot on oral contraceptive Use and breast cancer by mutation carries.

**Figure 3:**
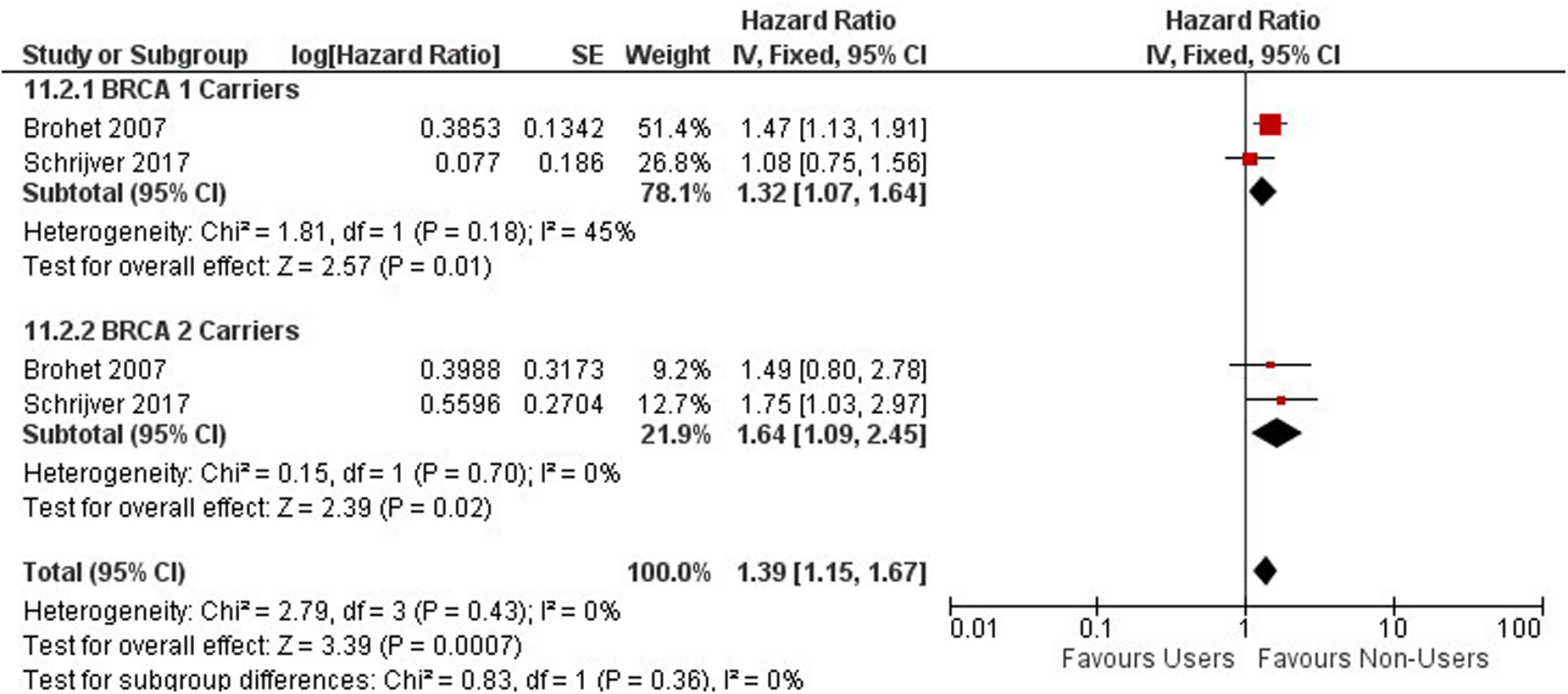
Forest plot on hormonal contraceptive methods and ovarian cancer.

**Figure 4:**
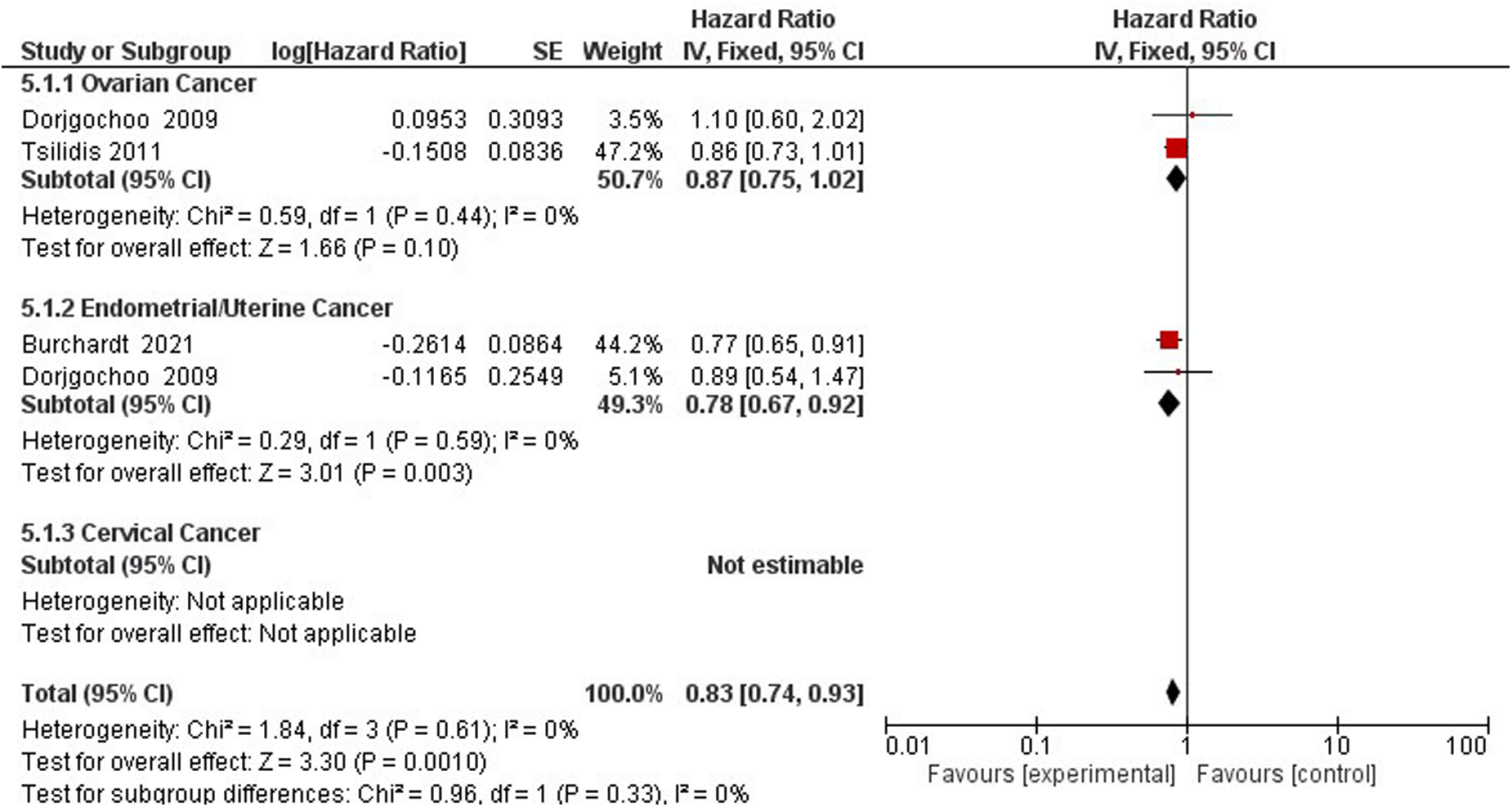
Forest plot on No-hormonal contraceptive methods and ovarian cancer.

**Figure 5:**
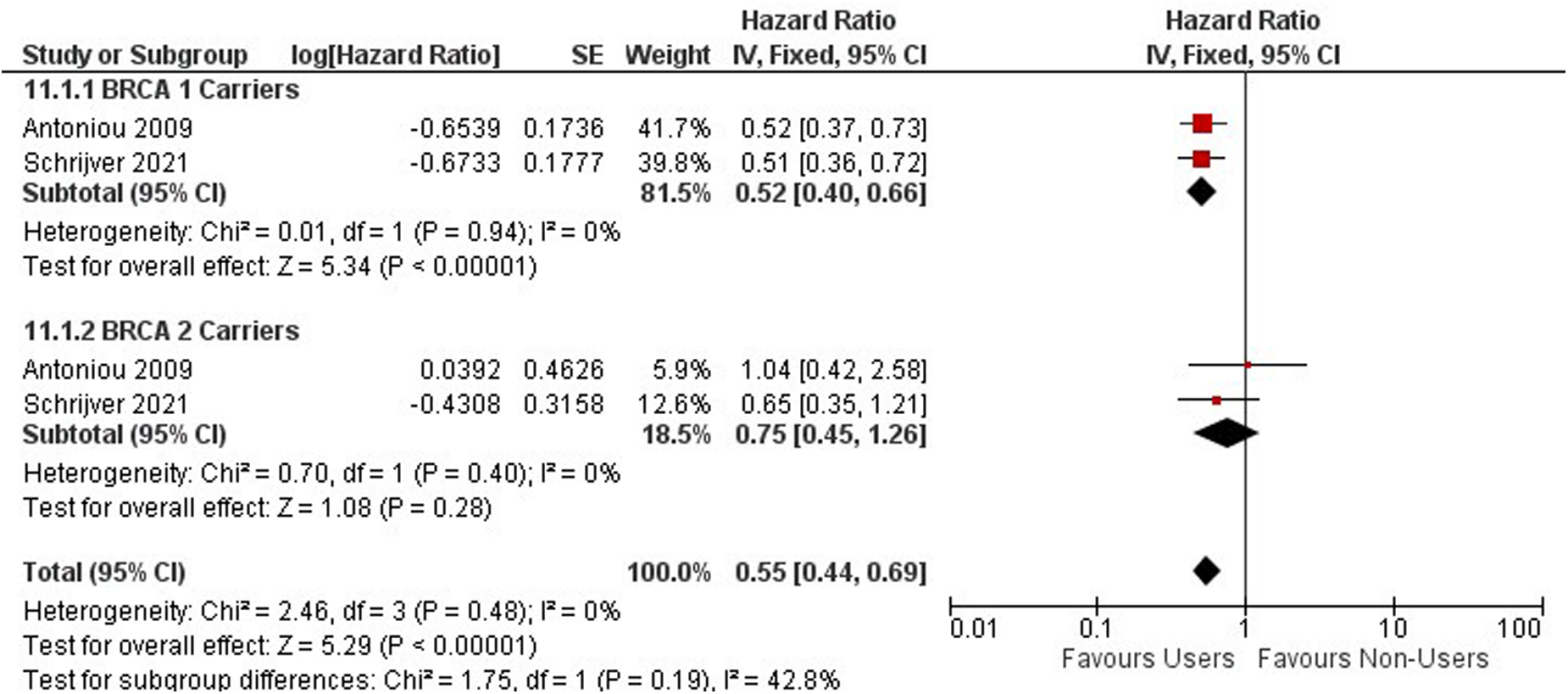
Forest plot on hormonal contraceptive Use and endometrial cancer.

**Figure 6:**
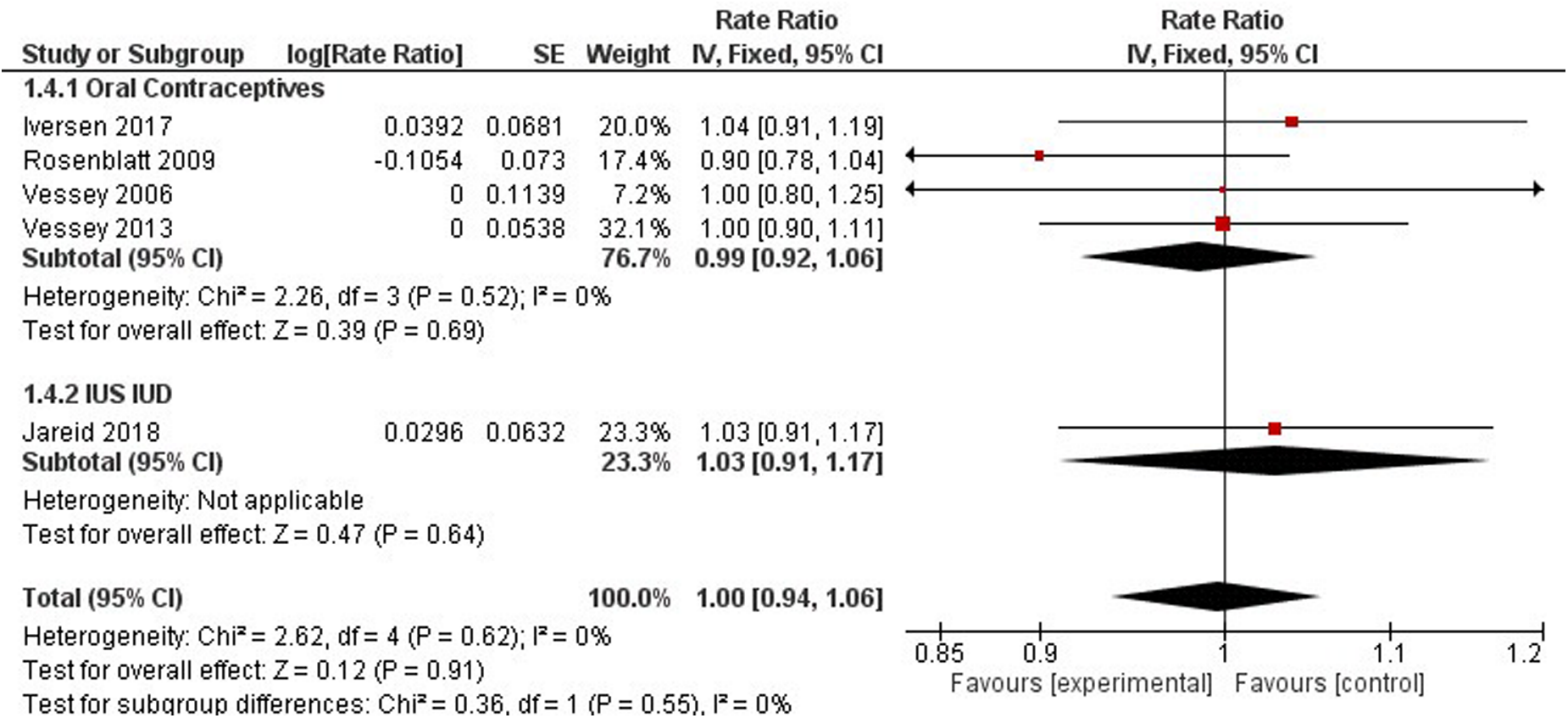
Forest plot on hormonal contraceptive Use and cervical cancer.

**Figure 7:**
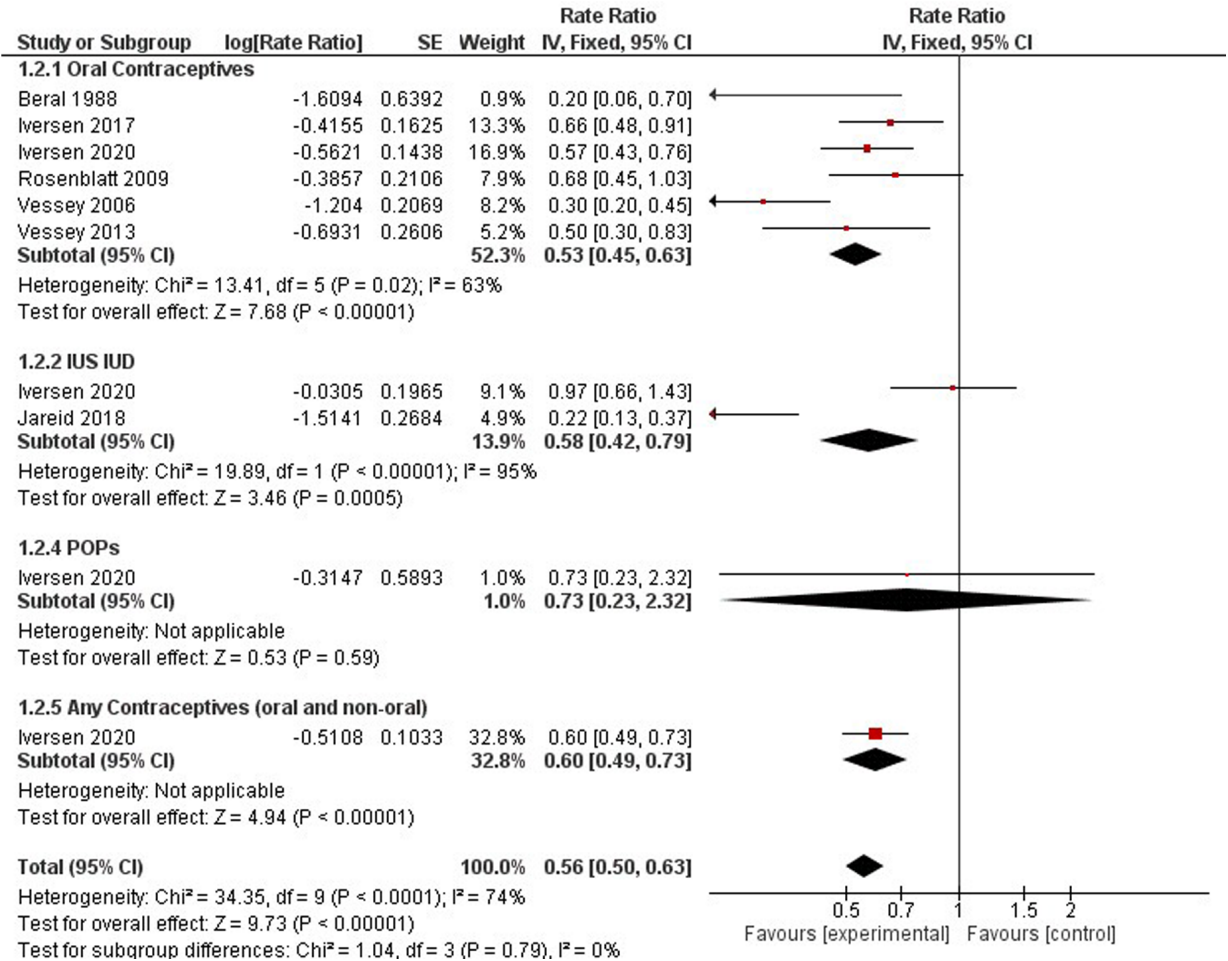
Forest plot on OCP use and gynecological cancer hazard.

**Figure 8:**
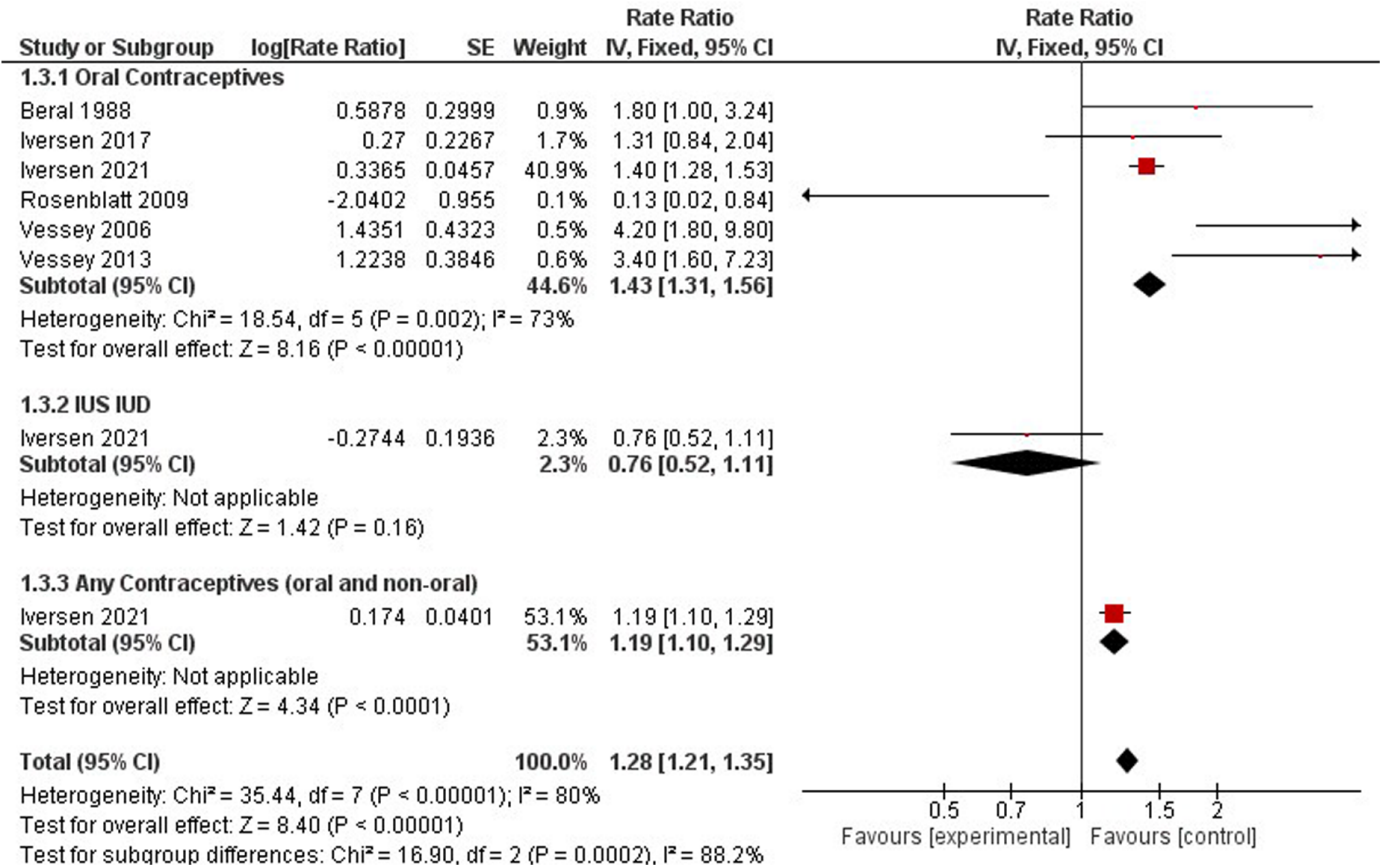
Forest plot on hormonal contraceptive Use and breast cancer.

**Figure 9:**
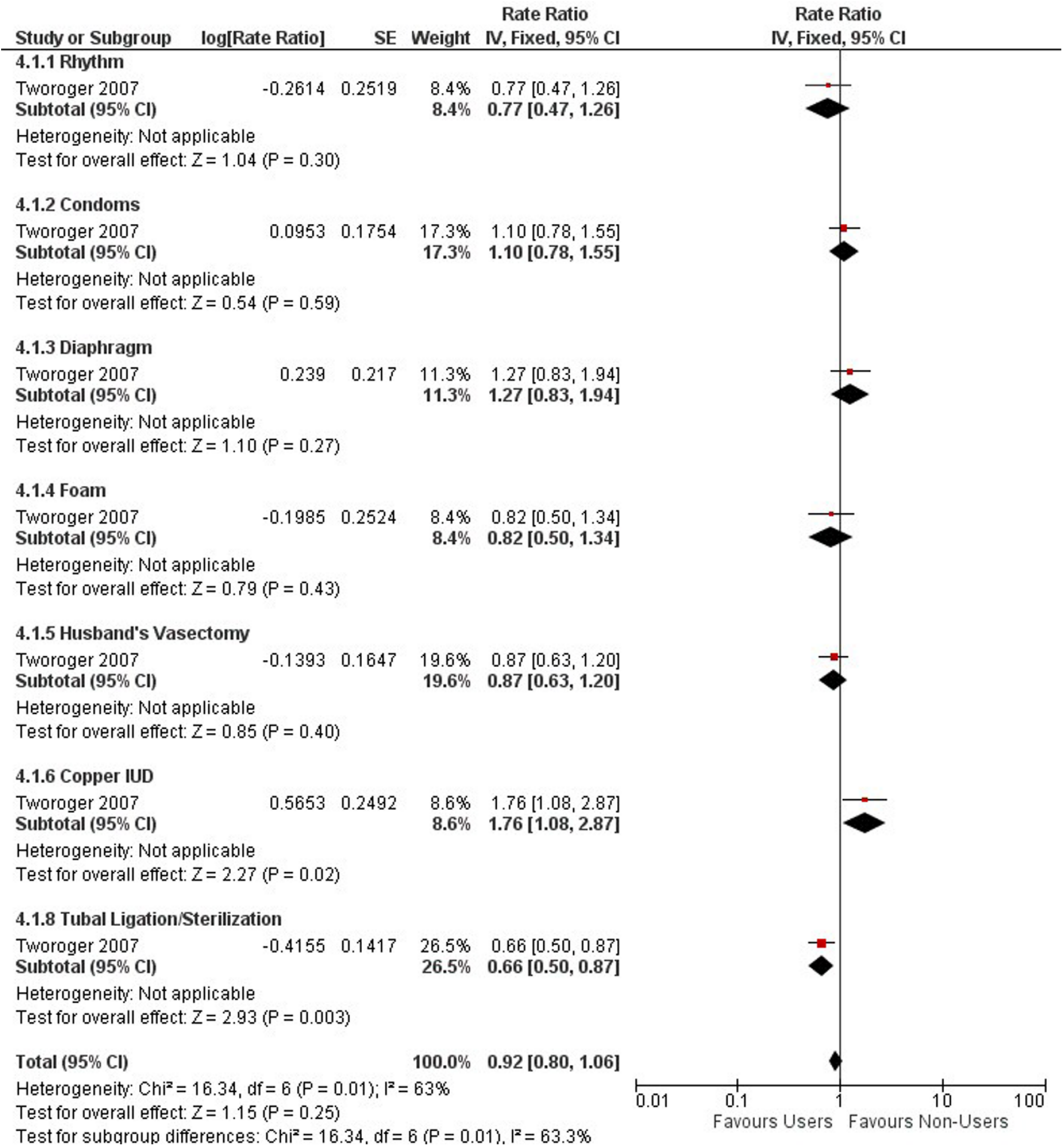
Forest plot on oral contraceptive Use and ovarian cancer by mutation carries.

**Figure 10:**
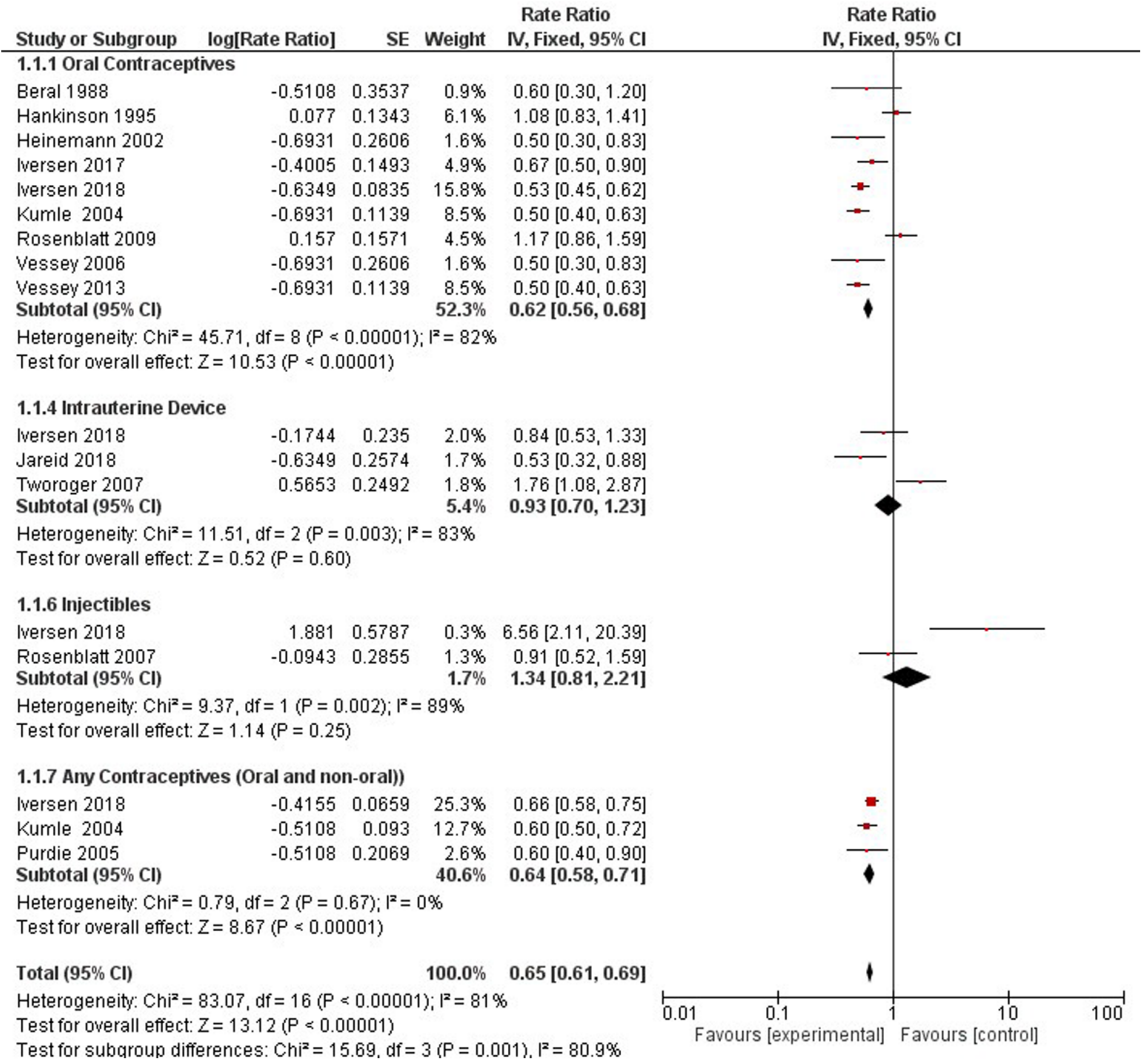
Forest plot on oral contraceptive Use and breast cancer by mutation carries.

