# Supplementary material for: Assessing the impact of contraceptive use on reproductive cancer risk among women of reproductive age—a systematic review
Source: Front Glob Womens Health. 2024 Nov 13;5:1487820. doi: 10.3389/fgwh.2024.1487820 (PMC11599208; doi:10.3389/fgwh.2024.1487820)
Supplement: Supplementary file 1 [file Supplementaryfile2.pdf]

Appendix 2 -Table S1. Characteristics of RCTS

Study (year)	Country	Study setting (public /private or rural/urban)	Study design	Sample size	Study aim	Population	Outcome (s) of interest	Intervention (RCTs)	Period of observation (weeks, months, years)	Findings (effect size and 95%CI)	Note
Fiascone 2018	United States	Private	RCT	76	Impact of depot medroxyprogesterone acetate on tumor glandular cellularity	Patients (>18 years) referred to the Women and Infants Program in Women's Oncology with new diagnosis of complex atypical hyperplasia, endometrial intraepithelial neoplasia, or grade-1 or -2 endometrioid adenocarcinoma of the uterus	Change in preoperative to postoperative tumor glandular cellularity. Defined as number of cells seen in one quarter of one high-powered field, averaged over 3 fields	38 patients received 400 mg DMPA injection and underwent surgery and 35 of them completed the FACT-En v4 survey prior to surgery. 38 patients received placebo injection and underwent surgery, and 32 of them completed the FACT-En v4 survey prior to surgery.	3 weeks	Scores from functional assessment of cancer therapy-endometrial survey: There were no significant differences between DMPA and placebo groups in any individual domain on the FACT-En instrument, nor were there differences in the total score (P = .600) or trial outcome index (P= .590)	Depot medroxyprogesterone acetate injection had significant tumor effect on type I endometrial cancers compared to placebo injection, while women waited for surgery
Hogewoning 2003	Netherlands	The colposcopy clinic of the Albert Schweitzer Hospital, Dordrecht, the Netherlands, from January 1995 to June 2002.	RCT	148	To evaluate CIN regression (2 consecutive “no cCIN” diagnoses at colposcopy) and HPV clearance (2 consecutive negative HPV tests were obtained)	125 women with CIN and their male sexual partners	Outcomes of interest were clinical regression of CIN at colposcopy and clearance of HPV.	condom use	Median follow-up time for women was 15.2 months (range 3.0-85.4).	Women in the condom group showed a 2-year cumulative regression rate of 53% vs. 35% in the noncondom group (p = 0.03). The 2-year cumulative rates of HPV clearance were 23% vs. 4%, respectively (p = 0.02). Although lower regression rates were found if women were HPV-positive and had > or =CIN2 lesions at baseline, effects of condom use were found both in women with CIN1 and in women with > or =CIN2 lesions. Condom use promotes regression of CIN lesions and clearance of HPV.	

Type your text

Appendix 2 -Table S2 . Characteristis of Observational Studies

Study (year)	Country	Study setting (public /private or rural/urban)	Study design	Sample size	Study aim	Population	Outcome (s) of interest	Exposure (observational studies)	Period of observation (weeks, months, years)	Findings (effect size and 95%CI)	Note
Antoniou 2009	European countries and Quebec	The International BRCA1/2 Carrier Cohort Study	Retrospective cohort study	3,319	To evaluate the effect of reproductive and hormonal factors on ovarian cancer risk in BRCA1 and BRCA2 mutation carriers.	Female carriers of pathogenic mutations in either BRCA1 or BRCA2, >18 years old	Ovarian cancer risk	Oral contraceptive	137,632 person-years	BRCA1 carriers who had ever used OC were at a significantly reduced risk of developing ovarian cancer (hazard ratio, 0.52; 95% confidence intervals, 0.37-0.73; P = 0.0002) and increasing duration of OC use was associated with a reduced ovarian cancer risk (P trend = 0.0004). Tubal ligation was associated with a reduced risk of ovarian cancer for BRCA1 carriers (hazard ratio, 0.42; 95% confidence intervals, 0.22-0.80; P = 0.008). The number of ovarian cancer cases in BRCA2 mutation carriers was too small to draw definitive conclusions	
Beral 1988	Britain	Private	Cohort study	47,000	Determine the relationship between oral contraceptive use and the incidence of, or mortality from cancers affecting the genital tract (cervix, uterus, ovary, vagina, vulva)	Women recruited through general practioners in the United Kingdom	Cancers of the cervix, uterus, ovary, vagina, vulva	Ever use of oral contraceptives	Women were followed from may 1968 to April 1987	The incidence of cervix cancers increased significantly with increasing duration of OC use. After more than 10 years of use, the incidence was more than 4 times that in never-users.<br><br>The incidence of other uterine and ovarian cancer declined with increasing duration of use, but the trends were not significant.	
Beral 1999	Britain	General Practices	Cohort study	46,000	Mortality associated with oral contraceptive use	Women recruited from general practices throughout the UK; users or non users of oral contraceptives; most are white, all are married	Mortality; Deaths related to cancers of the breast, cervix, uterus, and ovary. Deaths related to suicide	Oral contraceptives	Mean follow up time was 25 years.	Relative risk of mortality due to breast cancer 1.1 (95% CI = 0.8-1.4); Relative risk of mortality due to cervical cancer 1.7 (95% CI 0.9-3.2); RR due to uterine cancer 0.3 (95% CI 0.1 to 1.4); RR due to ovarian cancer 0.6 (95% CI 0.3-1.0); RR due to suicide 1.5 (95% CI 0.8-2.7)	
Brohet 2007	United Kingdom, Ireland, France and the Netherlands	Epidemiological Study of Familial Breast Cancer [EMBRACE]), France (Gene Etude Prospective Sein Ovaire [GENEPSO]) and the Netherlands (Gen en Omgeving studie van de werkgroep Hereditair Borstkanker Onderzoek Nederland [GEO-HEBON])	Retrospective cohort study	1,593	To examine the association between oral contraceptive use and risk of breast cancer among BRCA1/2 carriers	18 years or older women born after 1920, had been tested positive for BRCA1 or BRCA2 and informed of their mutation status.	Breast cancer risk	Oral contraceptive	65,180 person-years	There was an increased risk of breast cancer for BRCA1/2 mutation carriers who ever used oral contraceptives (adjusted hazard ratio [HR] =1.47; 95% CI, 1.16 to 1.87) compared to non-users.	
Burchardt 2021	United Sates	Multicenter	Cohort study	107,069	Endometrial cancer risk associated with oral contraceptive use	Women enrolled in the Nurses' Health Study II; Female nurses aged 25-42 years at recruitment	Endometrial Cancer; Subanalyses by histological subtype	Oral contraceptive use	A total of 107,069 women were followed from 1989 biennially until 2009. Cancer cases were tracked through 2017.	Relative to never users, ever OC users had lower endometrial cancer risk (HR 0.77 [95% CI 0.65–0.91], with lower risk associated with longer duration of use (e.g., >10 years, 0.43 [0.32–0.58]; p trend 5 years of OC use, versus never use, with a somewhat stronger association for more recent users (time since last use: ≤ 10 years, 0.43 [0.31–0.60]; > 10 years, 0.62 [0.50–0.78]	OC use was associated with a significantly lower risk of endometrial cancer relative to never users of oral contraceptives
Charlton 2014	United States	Mixed	Prospective cohort study	121,577	Determine the impact of oral conctraceptives on all casuses and cause specific mortality	Women recruited through the Nurse's Health Study. Married female registered nurses aged 30-55	All cause mortality, deaths organized into six major categories defined by ICD-8 codes. Two major categories of interest were cancer and violent or accidental deaths (suicide)	Oral Contraceptives (Ever Use, dosage not specified)	Women were followed for 36 years; Lifetime oral contraceptives use was recorded biennially from 1976 to 1982	Violent or accidental deaths were more common among ever users of oral contraceptives (HR 1.20, 95% CI 1.04, 1.37). Longer durations of use of oral contraceptives were associated with premature mortality due to breast cancer (P<0.0001) and decreased mortality rates of ovarian cancer (P=0.002). More time since last use of oral contraceptives was associated with violent/accidental deaths (P=0.005)	No association seen between ever use of oral contraceptives and all cause mortality.
Colditz 1994	United States	Nurses' Health Study	Prospective cohort study	166,755	To examine prospectively the risk for mortality among women who had ever used oral contraceptives compared with those who had never used oral contraceptives.	Female registered nurses aged 30 to 55 years in 1976	All deaths including a category for cancer (breast, endometrium, and ovary)	Oral contraceptive	1.3 million person-years	Mortality caused by breast cancer was not elevated among ever-users of oral contraceptives compared with never-users (relative risk, 1.07; CI, 0.86 to 1.34), and endometrial uterine cancer mortality showed an apparent but nonsignificant reduction (relative risk, 0.33; CI, 0.10 to 1.11).	
Dorjgochoo 2009	China	Population based - Urban	Prospective cohort study	66,661	Determine relationship between oral contraceptives and cancers of the reproductive system	Women aged 40-70 years, residing in 7 urban districts of Shanghai, China	Cancer of the breast, uterine body, and ovary	Ever use of oral contraceptives, ever use of IUD, ever use of tubal ligation	Median follow up of 7.5 years	Ever use of any contraceptive method was not associated with overall cancer risk [adjusted hazard ratio = 1.02, 95% CI, 0.92-1.12]. Longer duration of IUD use decreased risk for breast, theyoid, and lung cancers. Ever having a TS was associated with increased uterine body cancer [HR= 2.50, 95% CI, 1.47-4.25). No contraceptive method was found to be related to risk of ovarian cancer.	Various methods of contraception or reproductive patterns may impact development of cancer.
Faber 2013	Denmark	Population Based	Case-control study	554 women with ovarian cancer, 1,564 women with	Determine risk of ovarian cancer following use of estrogen and progestin	Women aged 35-79 years who were scheduled for an exploratory laparotomy or	Ovarian Cancer	Combined oral contraceptives or progestin-only pills for at least one month	Women interviewed more than a year following cancer diagnosis were excluded from analysis; more than 75% of participants	Exclusive use of combined oral contraceptives was associated with a statistically significantly decreased risk for ovarian cancer (OR = 0.68; 95 % CI 0.53–0.88), while no association was observed for the solitary use of	Study looks at time since first use, time since last use, duration of use
Falconer 2018	Sweden	Population Based	Cohort study	5,704,154	Association between tubal ligation and endometrial cancer risk	All women above 18 years of age in-between January 1, 1973 and December 31, 2009	Endometrial Cancer	Tubal Ligation	Women were followed between January 1, 1973 and December 31, 2009.	Tubal ligation was associated with a significantly reduced risk of endometrial cancer (HR 0.73, 95% CI 0.65-0.83). When considering effect of exposure time, tubal ligation was significantly associated with reduced risk of EC 10 years or more after surgery (HR 0.74, 95% CI 0.65-0.84).	Tubal ligation reduced risk of endometrial cancer.
Gabrick 2000	United States	Tumor Clinic of the University of Minnesota Hospital	Historical cohort Study	6,150	To determine whether the association between OC use and risk of breast cancer is influenced by family history of the disease.	Women with a family history of breast cancer	Relative risk (RR) of breast cancer associated with history of OC use by relationship to proband.	Oral contraceptive	Nearly 50 years	After accounting for age and birth cohort, ever having used OCs was associated with significantly increased risk of breast cancer among sisters and daughters of the probands (RR, 3.3; 95% confidence interval [CI], 1.6-6.7), but not among granddaughters and nieces of the probands (RR, 1.2; 95% CI, 0.8-2.0) or among marry-ins (RR, 1.2; 95% CI, 0.8-1.9).	
Graff-Iversen 2006	Norway	County level	Retrospective cohort study	20,282	Mortality associated with oral contraceptive use	Population based study included all inhabitants between 40-49 years old, and 10% of sample of 20-39 years old were invited	Mortality due to cancer (breast, cervix/corpus uteri, ovary)	EE 30-40 mg + progestin, EE 50 mg + progestin, progestin only, other/not specified OC	Follow up of 14 years among women who participated in survey	All cause mortality:<br>OC RR 0.45 (95% CI 0.28-0.99)<br>OC adjusted for age RR 0.89 (95% CI 0.52-1.87)<br>OC adjusted for age and education RR 0.87 (95% CI 0.46-1.65)	The finding of no overall difference in mortality in users of OC is in agreement with most other studies

Gross 1994	United States	The Cancer and Steroid Hormone (CASH) study, the Surveillance, Epidemiology, and End Results (SEER) Network, and published reports of epidemiological studies.	Population-based case-control study	4,817	To determine the effect of oral contraceptive (OC) use on the cumulative incidence of epithelial ovarian cancer from age 20-40, 20-50, and 20-55 years among four groups of women: positive family history, negative family history, parous, and nulliparous.	Women aged 20-54 years	Risk of epithelial Ovarian Cancer	Oral contraceptive	NA	OC use of 10 years is suggested to be protective against ovarian cancer among women with a family history of the disease. 5 year use of contraceptives among nulliparous suggested protective against ovarian cancer.	
Hankinson 1995	United States	The Nurses' Health Study	Prospective cohort Study	107,865	To examine the association between reproductive factors and ovarian cancer risk.	Female registered nurses in the US	New diagnosis of ovarian cancer in follow-up.	Self-reported oral contraceptive use in the survey questionnaires.	12 years	The age-adjusted relative risk associated with ever use of oral contraceptive was 0.86 (95% CI = 0.66-1.13), which in the multivariate model (controlling for age, parity, tubal ligation, age at menarche, age at menopause, smoking status, Quetelet's Index) changed to 1.08 (95% CI = 0.83-1.43).	
Hannaford 2010	United Kingdom	1400 general practitioners (GPs) throughout the United Kingdom between May 1968 and July 1969	Prospective cohort Study	Around 46,000	To see if the mortality risk among women who have used oral contraceptives differs from that of never users	Women who were using the pill and a similar number who had never used this method of contraception	Directly standardised adjusted relative risks between never and ever users for all cause and cause specific mortality	Oral contraceptive	Up to 39 years	Compared with never users, ever users of oral contraception had a significantly lower rate of death from large bowel/rectum (RR 0.62, 95% CI 0.46 - 0.83), uterine body (RR 0.43, 95% CI 0.21 - 0.88), and ovarian cancer (RR 0.53, 95% CI 0.38 - 0.72).	
Heinemann 2002	German	Cooperating physicians, drug stores, announcements in newspapers, magazines, brochures of health insurances, and contacts to friends or relatives of a respondent	Retrospective cohort Study	15,256	To provide information for the general population by describing the impact of OC use on public health.	Women 18 - 65 years in German	The association between historic OC use and the occurrence of tumours of the ovary and corpus uteri.	Self-reported never OC use in the survey questionnaires.	396,000 person-years	The adjusted relative risk [RR] for the occurrence of any uterine or ovarian cancer comparing users and non-users of OCs is 0.5 (95% confidence interval [95%CI]: 0.3 to 0.7).	
Husing 2016	Denmark, France, Germany, Greece, Italy, The Netherlands, Norway, Spain, Sweden and United Kingdom	23 study centers across 10 different countries	Prospective cohort Study	201,811	To develop a risk prediction model for endometrial cancer, combining the predictive capability from all of these risk factors	Women of convenience of volunteers agreeing to participate	Incident endometrial cancer cases.	Self-reported never OC use in the survey questionnaires.	The median follow-up of the study was 11.7 years (range 1–16 years)	The age-adjusted relative risk associated with ever use of oral contraceptive was 0.86 (95% CI = 0.66-1.13), which in the multivariate model (controlling for age, parity, tubal ligation, age at menarche, age at menopause, smoking status, Quetelet's Index) changed to 1.08 (95% CI = 0.83-1.43).	
Iversen 2007	United Kingdom	1400 general practitioners (GPs) throughout the United Kingdom between May 1968 and July 1969	Prospective cohort Study	5,602	To examine the relationship between tubal sterilization and subsequent all-cause death and the risk of any gynecologic and breast cancer in women in the United Kingdom	Sterilized women and nonsterilized women	Hazard ratios of all-cause death, gynecologic and breast cancer	Tubal sterilization	Sterilized women mean duration of follow-up, 338.9 ± 67.0 months; nonsterilized women mean duration of follow-up, 340.2 ± 66.1 months	Tubal sterilization was not associated with significantly altered risks of all-cause death.	
Iversen 2017	United Kingdom	1400 general practitioners (GPs) throughout the United Kingdom between May 1968 and July 1969	Prospective cohort Study	46,022	To examine the very long term cancer risks or benefits associated with the use of combined oral contraceptives, including the estimated overall life-time balance	Women who were using the pill and a similar number who had never used this method of contraception	Directly standardized rates of specific and any cancer for "ever" and "never" users of combined oral contraceptives	Combined oral contraceptive	Up to 44 years	Ever use of oral contraceptives was associated with reduced colorectal (incidence rate ratio, 0.81; 99% confidence interval, 0.66-0.99), endometrial (incidence rate ratio, 0.66; 99% confidence interval, 0.48-0.89), and ovarian (incidence rate ratio, 0.67; 99% confidence interval, 0.50-0.89).	
Iversen 2018	Denmark	Denmark, 1995-2014	Prospective, nationwide cohort study	1,879,227	To investigate the association between contemporary combined hormonal contraceptives (including progestogen types in combined preparations and all progestogen-only products) and overall and specific types of ovarian cancer.	All women living in Denmark aged 15-49 years	Relative risk of ovarian cancer among users of any contemporary combined hormonal contraceptives and by progestogen type in combined preparations and all progestogen-only products, including non-oral preparations.	Contemporary hormonal contraception	More than 21.4 million person years of observation (about 11.4 years per woman)	Compared with never users, reduced risks of ovarian cancer occurred with current or recent use and former use of any hormonal contraception (relative risk 0.58 (95% confidence interval 0.49 to 0.68) and 0.77 (0.66 to 0.91), respectively).	
Iversen 2020	Denmark	Denmark, 1995-2014	Prospective, nationwide cohort study	1,852,505	To examine the association between contemporary hormonal contraceptives and endometrial cancer risk in women younger than age 50.	Women living in Denmark aged 15–49 years through 1995–2014	Incidence rate ratios (RR) of endometrial cancer among ever, current or recent, and former users of any hormonal contraception.	Contemporary hormonal contraception	21.1 million person-years	Ever users of any hormonal contraception had a reduced premenopausal endometrial cancer risk compared with non-users; RR 0.60 (95% Confidence Interval 0.49 to 0.73).	
Iversen 2021	Denmark	Denmark, 1995-2014	Prospective, nationwide cohort study	1,853,542	To determine cervical cancer risk associated with contemporary hormonal contraceptives	Women living in Denmark aged 15–49 years through 1995–2014	Cervical cancer risks among different contraceptive user groups.	Contemporary hormonal contraception	>20 million person years	Ever users of any hormonal contraceptives compared to never users had a relative risk (RR) of 1.19 (95% confidence interval [CI] 1.10-1.29) of cervical cancer.	
Jareid 2018	Norway	Cohort from Norwegian Women and Cancer Study	Population-based prospective cohort study	104,318	To compare the adjusted risk of ovarian, endometrial, and breast cancer among LNG-IUS users and never users.	Women born between 1927 and 1965 randomly selected from the Norwegian Population Registry	The primary outcomes were epithelial ovarian cancer and endometrial cancer of the breast.	LNG-IUS (ever use, duration of use, age at first use, current use).	Women were enrolled between 1991–2007 and 2003–2006 and follow-up data was collected up to two times per participant after enrollment. Mean follow-up time was 12.5 (SD 3.7) years.	Compared to never users, LNG-IUS users had an RR of ovarian, endometrial, and breast cancer of 0.53 (95% CI 0.32, 0.88), 0.22 (95% CI 0.13, 0.40), and 1.03 (95% CI 0.91, 1.17), respectively.	Authors concluded LNG-IUS use is strongly associated with a decreased risk of ovarian and endometrial cancer compared to never users, and that there is no increased risk of breast cancer.
Karlsson 2021	United Kingdom	22 assessment centers across the United Kingdom	Case-control study	256,661	To determine time-dependent and long-term associations between oral contraceptive use and breast, ovarian, and endometrial cancer	Women born between 1939 and 1970 in the UK	Cumulative risk of cancer over the timespan of the study, as measured by the OR, and instantaneous risk, as measured by the HR	Oral contraceptive	2.01 million person-years for oral contraceptive users	The odds were lower among ever users, compared with never users, for ovarian cancer [OR = 0.72; 95% confidence interval (CI), 0.65–0.81] and endometrial cancer (OR = 0.68; 95% CI, 0.62–0.75). Increased odds were seen for breast cancer in women when limiting the follow-up to 55 years of age (OR = 1.10; 95% CI, 1.03–1.17), but not for the full timespan.	
Kumle 2004	Norway & Sweden	Population Based	Cohort study	103,551	Association between use of hormonal contraceptives and risk for invasive and borderline epithelial ovarian neoplasias	Women aged 30-49 years at enrollment in 1991/1992	Invasive and borderline epithelial ovarian neoplasias	Hormonal Contraceptive, including combined oral contraceptives (COCs) and progestins-only contraceptives (POPs; pills, injectable depot medroxy-progesterone acetate or levonorgestrel implants)	Women were recruited in 1991-1992 and followed until 2000.	Using the Cox proportional hazard models, ever having used hormonal contraceptives was associated with decreased relative risk of epithelial ovariance cancer (0.6, 95% CI 0.5-0.8)	

Loopik 2020	Netherlands	Cohort identified using the Dutch nationwide registry of histopathology and cytopathology between January 1, 2005 and December 31, 2009	Retrospective population-based cohort study	702,037	To evaluate the association between the type of contraceptive use and the development of cervical intraepithelial neoplasia grade III or worse (CIN3+).	Women aged 29 – 44 years attending the cervical cancer screening program with normal cytology between 2005 and 2009 identified from the Dutch Pathology Registry.	The primary outcome was cases of cervical cancer.	At least 5 years registered use of an oral contraceptive (OC) or IUD.	Median follow-up was 9.7 years.	Compared to never users, IUD use was associated with an increased risk of developing CIN3+ (RR 1.51, 95% CI 1.32–1.74), and OC use was associated with an increased risk of developing CIN3+ (RR 2.77, 95%CI 2.65–3.00) and cervical cancer (RR 2.06, 95%CI 1.5–2.79).	Authors concluded OC use and IUD use were associated with an increased risk of developing CIN3+. However, IUD use seems safer than an OC as the risk of developing CIN3+ and cervical cancer was higher for OC users.
Merritt 2015	European countries	Centres and national cohorts in European countries	Prospective cohort study	322,972	To provide evidence from a large European prospective study on how common reproductive factors may influence the long term health of women.	Women aged 25–70 years	Hazard ratios (HRs) and 95 % confidence intervals (CIs) for mortality	Oral contraceptive	A mean follow-up of 12.9 years	Among never/former smokers, we observed a lower risk of total cancer mortality in ever versus never users of OCs (HR = 0.91; CI, 0.85–0.98), while there was no association with OC use in participants who were current smokers at baseline (ever versus never users of OCs; HR = 1.00; CI, 0.90–1.12).	
Michels 2018	United States	Population-based recruitment of AARP members in 6 states and 2 metropolitan areas	Prospective Cohort study	196,536	To determine whether associations between duration of OC use and risk of specific cancers were modified by lifestyle characteristics.	AARP members who were between the ages of 50 and 71 years	Development of ovarian, endometrial, breast, and colorectal cancers.	Oral contraceptive	Around 16 years	For ovarian cancer, OC use–associated risk reductions strengthened with duration of use (long-term OC use [10 years] HR, 0.60; 95% CI, 0.47-0.76; P < .001 for trend) and were similar across modifiable lifestyle factors. Risk reductions for endometrial cancer strengthened with duration of use (long-term OC use HR, 0.66; 95% CI, 0.56-0.78; P < .001 for trend). Associations between OC use and breast and colorectal cancers were predominantly null.	
Miracle-McMahill 1997	United States	The Cancer Prevention Study II	Prospective Cohort study	396,114	To examine the relation between tubal ligation and ovarian cancer mortality	Women who were cancer free in 1982	Ovarian cancer mortality	Tubal ligation	9 years	Tubal ligation was significantly associated with a decreased risk of ovarian cancer mortality in an age- and race-adjusted Cox proportional hazards model (hazard ratio (HR) = 0.64, 95% confidence interval (CI) 0.42-0.96), and the results were essentially unchanged when controlling for potential ovarian cancer risk factors (HR = 0.68, 95% CI 0.45-1.03).	
Purdie 2005	Norway	National	Prospective Cohort study	103,551	Inverse association between use of hormonal contraceptives and risk of epithelial ovarian neoplasia	Women aged 30-49 years at enrollment, invited from national population registers.	Diagnosis of epithelial ovarian neoplasia	Hormonal contraceptives	Women were followed between 1991/1992 to the end of 2000	Relative risk for EON in ever users of hormonal contraceptives was 0.6 (95% CI 0.5-0.8). This was a similar value for current/past users, as well as users of COC and Progestin only preparations. Risk of ovarian cancer decreased with increasing duration of hormonal contraceptive use (p<0.0001)	Hormonal contraceptives reduce risk of epithelial ovarian neoplasia, compared to never users.
Rice 2014	United States	Nurses Health Study I/II	Prospective Cohort study	121,700 in cohort 1, 116,430 in cohort 2	Incidence of epithelial ovarian cancer related to contrapective use	Married US female nurses, aged 30-55 years at baseline and another cohort of US female nurses aged 25-42 years at baseline	Confirmed incident epithelial ovarian cancer	Tubal ligation, hysterectomy, unilateral oophorectomy	Follow up questionnaires were sent biennially between 1976 and 2010	Tubal ligation was associated with decreased risk of ovarian cancer (HR 0.76; 95% CI 0.64, 0.90). For nonserous tumors, the inverse association was stronger (HR 0.57; 95% CI 0.40, 1.02) and among women younger than 35 years at surgery (HR 0.67; 95% CI 0.49, 0.90). Hysterectomy was associated with decreased risk of ovarian cancer (HR 0.80; 95% CI 0.66, 0.97). Unilateral oophorectomy was associated with 30% lowered risk of ovarian cancer and did not differ by histological type (HR 0.70; 95% CI 0.53, 0.91)	Tubal ligation and hysterectomy reduce risk of ovarian cancer, especially for nonserous tumors and when surgeries are completed before 35 years
Robbins 2009	United States	Multicenter (atlanta, detroit, san fransico, seattle, connecticut, iowa, new mexico, and four urban counties of utah)	Case-control study	410 (N = 169 OCP use, N=241 never users)	To examine the influence of reproductive factors on ovarian cancer survival	Women with primary ovarian cancer diagnosed between December 1, 1980 and December 31, 1982 who had no history of ovarian cancer before the index diagnosis were eligible for the CASH study. longitudinal analysis of 410 women, ages 20 to 54 years, who participated in the 1980 to 1982 Cancer and Steroid Hormone study as incident ovarian cancer cases.	The influence of factors on ovarian cancer survival, the number of lifetime ovarian cycles	OCP	Median follow-up of 9.2 years	Of the reproductive factors examined, only age at menarche and number of lifetime ovulatory cycles (LOC) relative to age significantly predicted ovarian cancer survival. Risk for death was higher among women with highest number of LOC compared with those having fewest LOC (HR, 1.67; 95% CI, 1.20-2.33). Women with fewest LOC had the highest 15-year survival (56.7%; 95% CI, 47.8-64.6%), and women with the highest LOC had the poorest (33.3%; 95% CI, 25.3-41.5%). Women whose age at menarche was <12 years had a higher risk of death compared with women whose menses began at > or =14 years (HR, 1.51; 95% CI, 1.02-2.24).	
Rosenblatt 1996	Australia, Chile, China, Israel, Mexico, Philiphines, Thailand	Hospital based 9 centers in seven different countries	Case-control study	2,956	To examine the relationship between tubal ligation and hysterectomy and risk of ovarian cancer from a multinational study that was conducted largely in developing countries.	Women >15 years, born after 1925 or 1930 (depending on when hormonal contraceptives became available), living in the studies countries	Risk of ovarian cancer	Tubal ligation and hysterectomy	NA	A nonsignificant reduction in risk was observed for tubal ligation [odds ratio (OR), 0.72; 95% confidence interval (CI), 0.48-1.08] and hysterectomy (OR, 0.58; 95% CI, 0.26-1.27).	Data is stratified by parity, age at tubal ligation, time since sterilization, type of hysterectomy, histological typing of cancer. All data is adjusted for oral contraceptive use.
Rosenblatt 2007	China	Urban	Cohort study	267,400	Risk of all cancers among users of injectable contraceptives compared to nonusers	Female textile workers born between 1925 and 1958, working at one of 519 factories in the Shanghai Textile Industry Bureau (STIB), recruited between October 1989 and October 1991	Cancer; Breast, Ovary, Uterine Cervix, Uterine Corpus	Injectable contraceptives	Women were recruited between 1998-1991 and followed until July 2000.	RR for breast cancer, using never users as reference category: 0.90 (95% CI 0.71-1.13). RR for ovarian cancer, using never users as reference category: 0.91 (95% CI 0.52-1.60). RR for uterine cervix cancer 0.54 (95% CI 0.07-4.00). RR for uterine corpus cancer 0.61 (95% CI 0.42-0.87).	
Rosenblatt 2009	China	Rural	Cohort study	258,956	Risk of cancer following oral contraceptive use	Female textile workers in China who answered question on ever use of oral contraceptives	Cancer (all types); Ovarian, Breast, Uterine (Cervical, Corpus)	Ever use of oral contraceptives.	Women were recruited between 1998-1991 and followed until July 2000.	No increased risk of all cancers combined or breast, colon, gall bladder, liver, lung, ovarian, pancreatic, rectal, stomach, thyroid, or cervical for people who use oral contraceptives. Significant reduction in risk of endometrial cancer (uterine corpus) for users of contraceptives Decreasing risk with increasing duration of use<br>See: non users = 236 cases/1982639 person years<br>1-11 month users = 14 cases/131,423 person years<br>12+ month users = 13 cases/213,962 person years	

Schrijver 2018	Countries in Europe, America, and Oceania	Three large cohorts: the International BRCA1/2 Carrier Cohort Study (IBCCS), the Kathleen Cuningham Foundation Consortium for Research Into Familial Breast Cancer (kConFab) Follow-Up Study, and the Breast Cancer Family Registry (BCFR)	Cohort study	9,839	To analyze the association between the use of oral contraceptive preparations (OCPs) and breast cancer (BC) risk in the first prospective analysis.	Women had a pathogenic mutation in either BRCA1 or BRCA2 between age 18 and 80 years	Risk of developping breast cancer and ovarian cancer	Oral contraceptive	The mean follow-up years of the prospective cohort, the retrospective "left-truncated" cohort, and the retrospective "full-cohort" was 4.2, 3.0, and 40.1 years in BC patients and 5.6, 4.3, 38.7 years in non-BC participants respectively.	From the prospective cohort analysis, ever OCP use was not associated with BC risk (HR = 1.08, 95% confidence interval [CI] = 0.75 to 1.56). In contrast, from the left-truncated and full-cohort retrospective analyses, an association between ever OCP use and BC risk (HR = 1.26, 95% CI = 1.06 to 1.51, and HR = 1.39, 95% CI = 1.23 to 1.58, respectively) was found.	
Schrijver 2021	European countries	The International BRCA1/2 Carrier Cohort Study (IBCCS)	Retrospective Cohort study	6,434	To investigate in more detail the associations of various characteristics of oral contraceptive use and risk of ovarian cancer	Women carrying a pathogenic or likely pathogenic germline mutation in BRCA1 or BRCA2.	Risk of developping ovarian cancer	Oral contraceptive	The mean person-year of BRCA1 carrier and BRCA2 carrier was 3.2 and 2.9 person-years in ovarian cancer patients, and 4.2 and 4.4 person-years in non-ovarian-cancer participants.	Duration of oral contraceptive use proved to be the prominent protective factor of ovarian cancer (compared with <5 years: 5-9 years [hazard ratio, 0.67; 95% confidence interval, 0.40-1.12]; >10 years [hazard ratio, 0.37; 95% confidence interval, 0.19-0.73]; P=.008).	
Shafrir 2017	United States	The Nurses' Health Study II (NHSII)	Prospective Cohort study	110,929	To assessed OC use, including type and dose, and ovarian cancer risk among women born between 1947 and 1964 using more recent formulations.	US female nurses, aged 25–42	Risk of developping ovarian cancer	Oral contraceptive	2,178,679 person-years	Compared to never use, we observed an increased risk of ovarian cancer with ≤6 months of OC use (HR 1.82; 95% CI 1.13–2.93) but a non-significant 57% (95% CI 0.18–1.03) decreased risk with ≥15 years of OC use. The increased risk among short-term users (≤1 year) was restricted to OCs containing mestranol (HR 1.83; 95% CI 1.16–2.88) and first-generation progestin (HR 1.72; 95% CI 1.11–2.65).	
Sponholtz 2018	United States	The Black Women's Health Study (BWHS)	Prospective Cohort study	47,555	To contribute to the body of evidence regarding use of exogenous hormones and risk of endometrial cancer in black women	Black women ages 21-69	Risk of developping endometrial cancer	Oral contraceptive	18 years	Compared with never use, > 10 years' duration of OC use was associated with lower risk (multivariable IRR = 0.45, 95% CI, 0.27–0.74), but risk was higher among current users of estrogen-only (IRR = 3.78, 95% CI, 1.69–8.43) and estrogen plus progestin FMH (IRR = 1.55, 95% CI, 0.78–3.11).	
Syrjanen 2006	The former Soviet Union	6 different outpatient clinics in three New Independent States (NIS) of the former Soviet Union	Prospective Cohort Study	3,097	To analyse the risk estimates for OC users in order to develop several intermediate end-point markers in cervical carcinogenesis.	Women patients from cervical cancer screening, gynaecology outpatient clinics, and STD clinics	a) exposure to HR-HPV; b) progression to high-grade cervical intraepithelial neoplasia (CIN2/3 and HSIL); and c) persistence/clearance of HR-HPV and cytological abnormalities	Oral contraception	Median FU 16.7 months	When analysed separately for HPV-positive and HPV-negative women, use of OC was not a significant predictor of CIN2/3 in either group; OR=0.98 (95% CI 0.53-1.82) and OR=0.92 (95% CI 0.10- 8.85), respectively.	
Tsilidis 2011	Denmark, Italy, The Netherlands, Norway, Spain, France, Germany, Greece, Sweden and the UK	Multicountry population based study	Prospective cohort study	327,396	To examine the associations of oral contraceptive use and reproductive factors with ovarian cancer risk in the European Prospective Investigation into Cancer and Nutrition.	Women living in the studied countries	Hazard ratios (HRs) and 95% confidence intervals (CIs) of ovarian cancer	Oral contraceptive	2.9 million person-years of follow-up, over an average of 9 years	Compared with never users of oral contraceptives, ever users had a significantly lower risk of ovarian cancer in the age and centrestratified model (HR, 0.84; 95% CI, 0.72–0.98), and that remained very similar after additional adjustment for smoking status, BMI, unilateral ovariectomy, simple hysterectomy, menopausal hormone therapy, age at menarche, age at menopause and number of full-term pregnancies (HR, 0.86; 95% CI, 0.73– 1.00)	
Tworoger 2007	United States	The US Nurses' Health Study	Prospective Cohort Study	107,900	To examine ovarian cancer risk in relation to duration and time since last oral contraceptive use, and in relation to other contraceptive methods and infertility among participants in the Nurses' Health Study.	Female registered nurses	Risk of epithelial ovarian cancer	OCP, tubal ligation, rhythm method, diaphragm, condoms, intrauterine device, foam, spousal vasectomy	28 years of follow-up	Duration of oral contraceptive use was inversely associated with risk (p-trend = 0.02), but no clear trend was observed for years since last use. However, for women using oral contraceptives for >5 years, the rate ratio for ovarian cancer for ≤ 20 years since last use was 0.58 (95% confidence interval (CI): 0.39, 0.87), with no association found for >20 years since last use (rate ratio (RR) = 0.92, 95% CI: 0.61, 1.39). Tubal ligation (RR = 0.66, 95% CI: 0.50, 0.87) was associated with decreased ovarian cancer risk, whereas intrauterine device use (RR = 1.76, 95% CI: 1.08, 2.85) and infertility (RR = 1.36, 95% CI: 1.07, 1.75) were associated with an increased risk.	
Vaisy 2014	Iran	Obstetrics and gynecology clinics of Imam Khomeini and Imam Hussein Hospitals (Urmia, Iran)	Case-control study	128 iranian patients age 25-60 y/o w/ cervical cancer, 235 breast cancer and equal numbers of controls	To investigate the relationship between the incidence of cervical and breast cancers and oral contraceptive use (looked at incidence of cervical and breast cancers)	The subjects were all Iranian, Muslim, married, and 20-65 years old.	Incidence of cervical and breast cancer following use of contraceptive pills	Contraceptive pills	NA	The OR of developing cervical cancer following the use of Oral contraceptive (OR=3.072; 95%CI: 1.84-5.11). Use of contraceptive pills doubled the odds for affliction with breast cancer (OR=2.11; 95%CI: 1.44-3.08). The current study did not show any significant relation between age at first use of oral contraceptives and incidence of cervical cancer. Those who use OCP for greater than 97 months is at a significantly increased odds for cervical cancer OR 5.2 (2.28-11.8) compared to never users. In the case group, there was a significant difference between the initiation of oral contraceptives before and after the age of 25 years. The odds of developing the disease increased by starting the pills at an age older than 25 years. The OR of suffering from breast cancer was 1.99 (95%CI: 1.2-3.3) in those who had started oral contraceptives after the age of 25 years and increased to 6.47 (95%CI: 2.46-17.04) in subjects who had started the pills after 30 years of age.	The current study failed to establish a significant relationship between the incidence of cervical cancer and type of Oral contraceptive
Vessey 2006	United Kingdom	17 family planning clinics in England and Scotland	Cohort study	17,032	To examine cancer incidence in relation to oral contraceptive (OC) use	Women at ages 25 – 39 years	Cancer incidence	Oral contraceptive	540,000 woman-years	Breast cancer incidence was unrelated to duration of OC use (ever vs never users) (RR 1.0, 95% CI 0.8 –1.1). Cervical cancer showed a strong positive association with duration of OC use (RR 4.2, 95% CI 1.8 –12.0). Both uterine body and ovarian cancer were strongly negatively associated with OC use; the ever-used to never-used comparisons yielded rate ratios of 0.3(0.2 –0.6) and 0.5(0.3 –0.7), respectively.	
Vessey 2010	England and Scotland	Multicenter	Prospective cohort study	17,032	Factors impacting mortality	Women aged 25-39 years, married, white, British, willing to cooperate and either a current OC user, IUD user, or diapraghm user of at least 5 months	Mortality; Breast Cancer, Cervical Cancer, other Uterine Cancer, Ovarian Cancer	Oral contraceptives, diaphragm, or IUD	Women were recruited between 1968-1974 and followed until March 2009. Women were followed until reaching age 45; after age 45 they were allocated to one of 3 groups base on OC use (never, >8 years, <8 years). Total of 602,700 woman years.	Shows OC use and mortality from breast cancer to be completely unrelated (RR 1.0, CI 0.8-1.2). Cervical cancer mortality, however, is much increased in OC users (RR 7.3), but the CI is extremely wide (1.2–305) and the number of deaths (19) small. Other uterine cancer (mostly endometrial) and ovarian cancer have significantly low RRs in OC users (0.3, CI 0.1–0.8 and 0.4, CI 0.3–0.6 respectively). There are no other significant differences but considering all cancers together, the RR is slightly reduced in OC users, the difference being of borderline significance (RR 0.9, CI 0.8–1.0)	

Vessey 2013	United Kingdom	Private (Family Planning clinics in England)	Prospective Cohort Study	17,032	The association of oral contraceptives and cancer incidence	Women aged 25-39, married, White, British, willing to cooperate and either a current OC user of at least 5 months or a current diaphragm user or IUD user	Incidence of cancer	Oral contraceptives, IUD, diapraghm	602,700 Woman-years of observations	The rate ratio (RR) comparing ever users of OCs with never users was 1.0 [95% CI 0.9-1.1). Only two cases of cervical cancer have been added since our last report (total: 61 cases); the RR comparing ever use with never use is now 3.4 (95% CI: 1.6–8.9). The risk of this disease increases sharply with duration of OC use and declines steadily with interval since last OC use. OC use protects against both uterine body cancer (124 cases) and ovarian cancer (143 cases). The RRs comparing ever use with never use were 0.5 (95% CI: 0.3–0.7) and 0.5 (95% CI: 0.4–0.7), respectively. Protection against both these cancers increased with duration of OC use and waned with interval since last use, but an effect was still present 28 or more years after discontinuation.	Oral contraceptive use had no effect on nonreproductive cancers or on breast cancer. The risk of cervical cancer was increased and that of uterine body cancer and ovarian cancer was decreased by OC use. all effects increased with duration of use and declined with interval since last use.
Westreich 2014	South Africa	An adult HIV outpatient clinic in a teaching hospital affiliated with the University of Witwatersrand in Johannesburg	Cohort longitudinal observational study	594	To investigate whether use of hormonal contraception was associated with increased incidence or progression of cervical disease	HIV infected women ages 18 to 65 years	Risk of cervical cancer	Hormonal contraception	A mean of 551 days	The effect of any exposure to any hormonal contraception on incidence or progression of SIL was 1.11 (95% CL 0.62, 1.97).	
Winer 2016	United States	Clincal Centers	Secondary analysis of cohort study	76,483 (53,737 women from OS and 22,746 women from DM)	Risk of endometrial cancer following tubal ligation	Women between ages of 50-79, post menopausal, and planning to reside in the same area for at least 3 years	Endometrial Cancer	Bilateral tubal ligation	Mean followup time was 11.4 years	A total of 1137 women were diagnosed with incident endometrial cancer (972 type I and 128 type II) during a mean follow-up of 11.3 years. Overall, 14,499 (19%) women had undergone BTL. There were no statistically significant associations noted between BTL and age at BTL for type I or type II endometrial cancers suggesting that patients undergoing this popular birth control method likely do not have an associated change in their baseline risk for endometrial cancer. The adjusted model adjusted for estrogen use, oral contraceptives, age, race, bmi, etc.<br>Type 1 ADJUSTED FOR AGE, REGION, RACE, BMI ETC HR 0.97 (95% CI; 0.811,1.17, P=0.780)<br>Type 2 HR 1.14 (95% CI 0.69,1.90, P=0.601)	Post menopausal women are in the study, but data is divided into participants who received treatment before 40 years old.
Xia 2021	Mixed	Public	Case-control study	14,199 BRCA 1 or BRCA2 carriers	Ovarian cancer risk among BRCA1 and BRCA2 mutation carriers who use contraceptives	All women who had sought genetic testing due to a personal or family history of breast and/or ovarian cancer	Ovarian cancer	Any contraceptive use	NA	Cases were less likely to have a history of oral contraceptive use (44.66% vs. 52.68%; P < 0.0001) and implant use (0.35% vs. 1.38%; P = 0.001) than controls. Compared to never users of any hormonal contraceptive, women who used any type of hormonal contraceptive had a significant 38% lower risk of developing ovarian cancer according to both the unadjusted and adjusted models (OR = 0.62; 95% CI 0.53–0.73 and OR = 0.62; 95% CI 0.52–0.75 respectively).<br>OC = 34% reduced risk<br>Implants =70% reduced risk and Injections= 63% reduced risk, however small sample size so use caution<br>IUD= reduced but not significant risk by 32%	
Zondervan 1996	United Kingdom	17 large family planning clinics in England and Scotland	Nested case-control study	310 cases under the age of 45 years, married, white, and british	Follow women to see if they develop Invasive, In Situ, Dysplasia, All Cervical Neoplasia	women from the Oxford Family Planning Associatoin contraceptive study; women diagnosed under 45 with invasive carcinoma (n=33), carcinoma in situ (n=121) or dysplasia (n=159). controls were randomly selected from among cohort members and matched to cases on exact year of birth and clinic attended at recruitment of study	The association between oral contraceptions and cervical neoplasia	Oral contraceptives	12 years of f/u (after initial 10 year f/u of cohort of 17,000 women) = total of 22 years of f/u	Ever users of OCs had a slightly elevated OR for all types of cervical neoplasia combined (OR = 1.40, 95% CI 1.00-1.96). Odds ratios were highest for invasive carcinoma (OR = 4.44, 95% CI 1.04-31.6), intermediate for carcinoma in situ (OR = 1.73, 95% CI 1.00-3.00) and lowest for dysplasia (OR = 1.07, 95% CI 0.69-1.66). Among current or recent users, ORs for all types of cervical neoplasia combined were 3.34 (95% CI 1.96-5.67) for 49-72 months of use, 1.69 (95% CI 0.97-2.95) for 73-96 months and 2.04 (95% CI 1.34-3.11) for 97 or more months.	

Appendix 2 -Table S3. Dawn and Black Quality Assessment

Author, year	Reporting	External validity	Internal validity		Total score
			Bias	Confounding	
Antoniou 2009	4	2	4	3	13
Beral 1988	2	0	2	3	7
Beral 1999	4	1	4	3	12
Brohet 2007	6	2	4	3	15
Burchardt 2021	6	1	4	3	14
Charlton 2014	6	2	5	3	16
Colditz 1994	6	2	4	3	15
Dorjgochoo 2009	5	2	4	3	14
Faber 2013	6	1	3	3	13
Falconer 2018	6	2	4	3	15
Gabrick 2000	6	1	4	2	13
Graff-Iversen 2006	4	2	3	2	11
Gross 1994	6	2	2	2	12
Hankinson 1995	5	2	5	3	15
Hannaford 2010	6	1	5	3	15
Heinemann 2002	4	1	5	3	13
Husing 2016	6	2	4	2	14
Iversen 2007	5	1	4	3	13
Iversen 2017	6	1	5	3	15
Iversen 2018	6	2	5	2	15
Iversen 2020	6	2	5	2	15
Iversen 2021	6	2	5	2	15
Jareid 2018	6	1	3	2	12
Karlsson 2021	4	0	4	3	11
Kumle 2004	6	1	3	3	13
Loopik 2020	6	0	5	1	12
Merritt 2015	6	0	5	1	12
Michels 2018	5	2	4	3	14
Miracle-McMahill 1997	6	2	5	3	16
Purdie 2005	1	1	1	2	5
Rice 2014	4	1	4	2	11
Robbins 2009	5	2	4	3	14
Rosenblatt 1996	4	1	4	3	12
Rosenblatt 2007	4	1	4	3	12
Rosenblatt 2009	5	2	5	3	15
Schrijver 2018	5	2	4	3	14
Schrijver 2021	4	2	4	3	13
Shafrir 2017	5	1	5	3	14

Sponholtz 2018	6	2	5	3	16
Syrjanen 2006	5	1	3	3	12
Tsilidis 2011	6	2	4	2	14
Tworoger 2007	6	2	4	3	15
Vaisy 2014	4	1	3	0	8
Vessey 2006	5	2	4	3	14
Vessey 2010	5	2	4	3	14
Vessey 2013	5	2	4	3	14
Westreich 2014	6	2	5	2	15
Winer 2016	6	2	5	3	16
Xia 2021	6	2	3	3	14
Zondervan 1996	5	1	2	2	10
