# Supplementary material for: Assessing the impact of contraceptive use on reproductive cancer risk among women of reproductive age—a systematic review
Source: Front Glob Womens Health. 2024 Nov 13;5:1487820. doi: 10.3389/fgwh.2024.1487820 (PMC11599208; doi:10.3389/fgwh.2024.1487820)
Supplement: Supplementary file 2 [file Supplementaryfile3.docx]

**Appendix – figures**


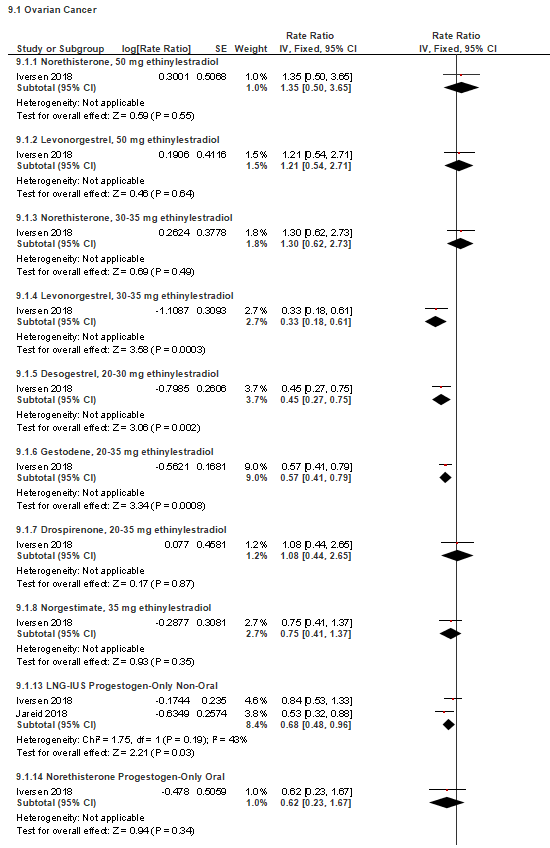


Appendix 3- **Figure S1 . Forest Plot on Hormonal Contraceptive Dose Regimens and Ovarian Cancer**


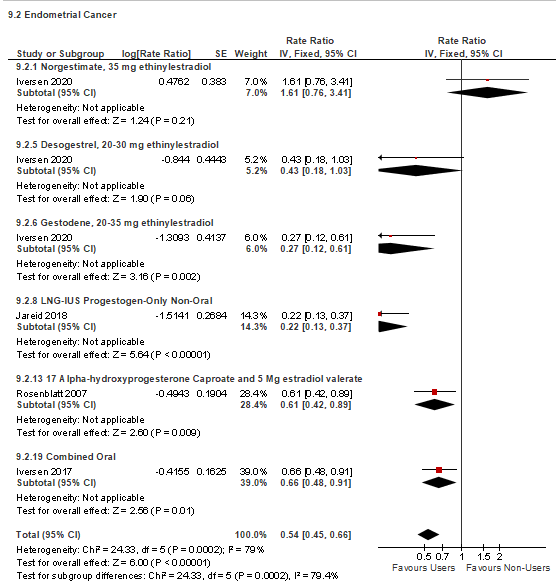


Appendix 3- Figure S2**. Forest Plot on Hormonal Contraceptive Dose Regimens and Endometrial Cancer**


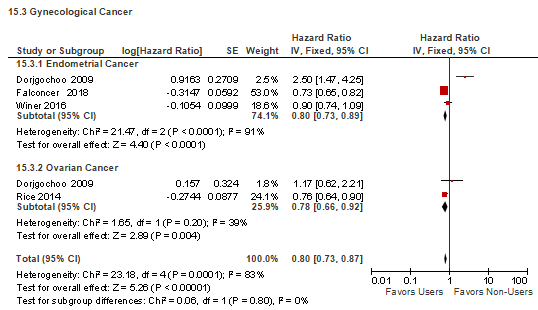


Appendix 3- **Figure S3. Forest Plot on Tubal Ligation and Cancer Outcomes**


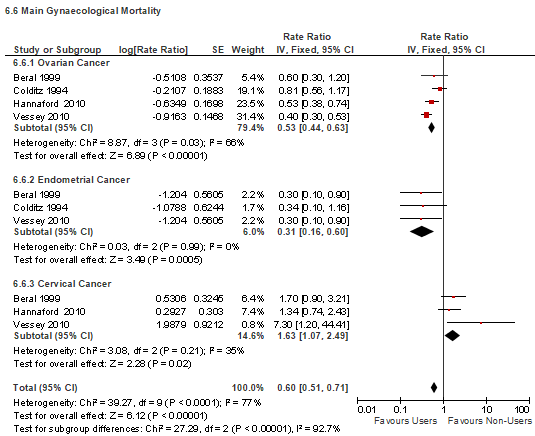


**Appendix 3- Figure S4. Forest Plot on Hormonal Contraception and Gynecological Cancer Mortality**


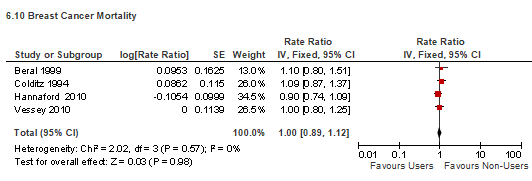


**Appendix 3- Figure S5. Forest Plot on Hormonal Contraceptive Use and Breast Cancer Mortality**


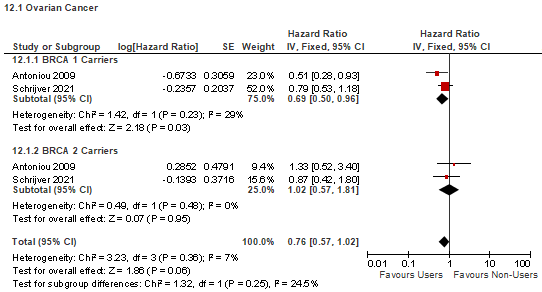


Appendix 3- **Figure S6. Forest Plot on Short Term (<5 years) Contraceptive Use and Ovarian Cancer**


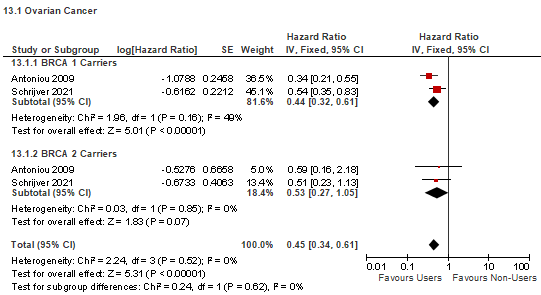


Appendix 3- Figure S7**. Forest Plot on Contraceptive Use Between 4-9 years and Ovarian Cancer**


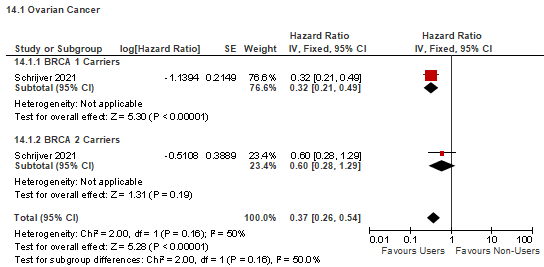


Appendix 3- **Figure S8. Forest Plot on Longer Term (> 10 years) Contraceptive Use and Ovarian Cancer**


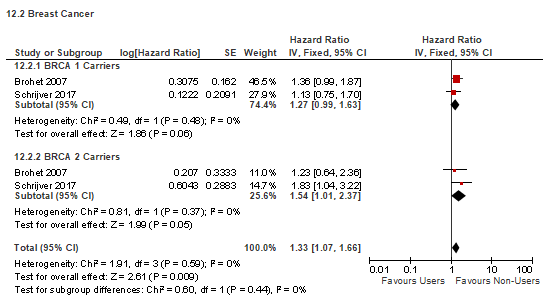


Appendix 3- **Figure S9. Forest Plot on Short Term (<5 years) Contraceptive Use and Breast Cancer**


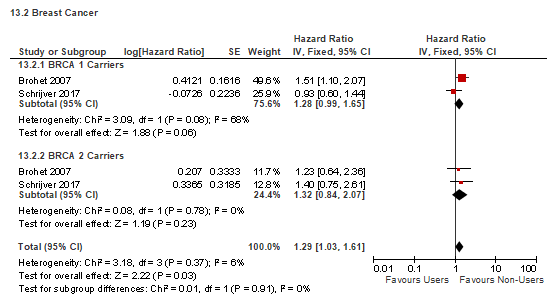


Appendix 3- **Figure S10. Forest Plot on Contraceptive Use Between 4-9 years and Breast Cancer**


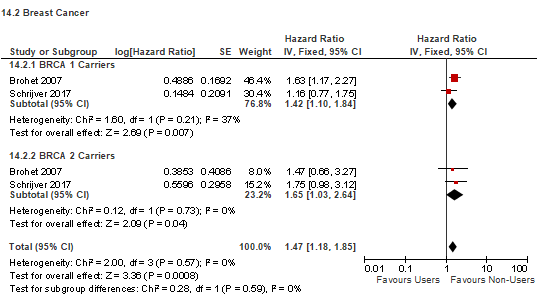


Appendix 3- **Figure S11. Forest Plot on Longer Term (> 10 years) Contraceptive Use and Breast Cancer**


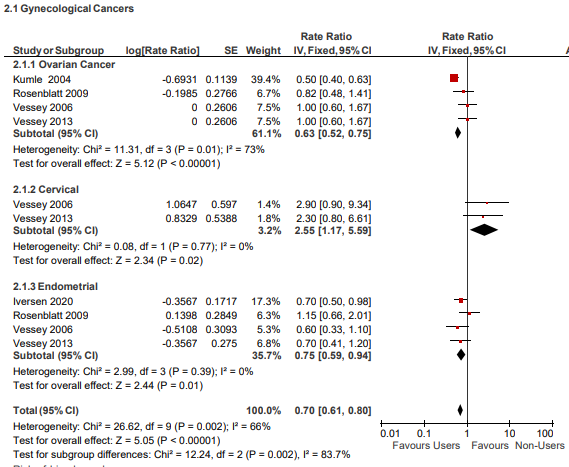


Appendix 3- **Figure S12. Forest Plot on Short Term Contraceptive Use and Gynecological Cancer, duration of use**


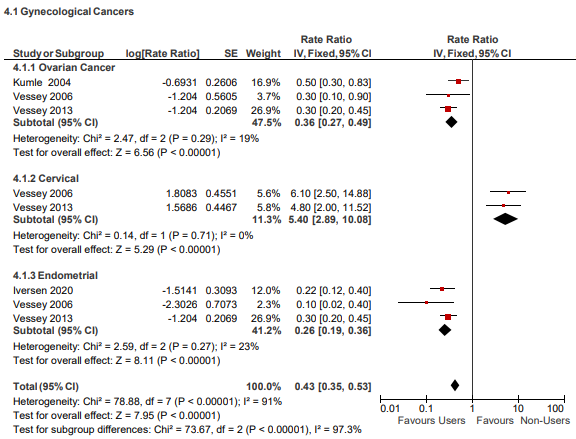


Appendix 3- **Figure S13. Forest Plot on Long Term Contraceptive Use and Gynecological Cancer, duration of use**


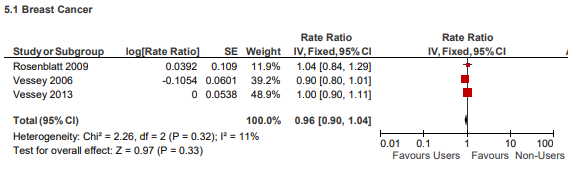


**Appendix 3- Figure S14. Forest Plot on Short Term Contraceptive Use and Breast Cancer**


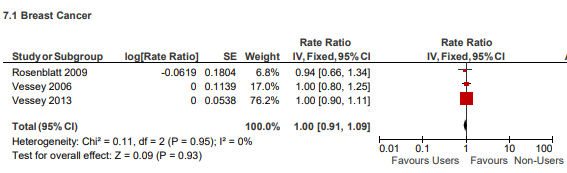


Appendix 3- **Figure S15. Forest Plot on Long Term Contraceptive Use and Breast Cancer**
